# A Comprehensive Evaluation of Cross-Omics Blood-Based Biomarkers for Neuropsychiatric Disorders

**DOI:** 10.3390/jpm11121247

**Published:** 2021-11-24

**Authors:** Weichen Song, Weidi Wang, Zhe Liu, Wenxiang Cai, Shunying Yu, Min Zhao, Guan Ning Lin

**Affiliations:** 1 School of Biomedical Engineering, Shanghai Jiao Tong University, Shanghai 200030, China; goubegou@sjtu.edu.cn (W.S.); wwd-swxx@foxmail.com (W.W.); liuzm@sjtu.edu.cn (Z.L.); caiwenxiang@sjtu.edu.cn (W.C.); 2 Shanghai Key Laboratory of Psychotic Disorders, Shanghai 200030, China; yushuny@yahoo.com (S.Y.); drzhaomin@gmail.com (M.Z.)

**Keywords:** peripheral biomarker, neuropsychiatric disorder, mendelian randomization, diagnostic model, prognosis

## Abstract

The identification of peripheral multi-omics biomarkers of brain disorders has long been hindered by insufficient sample size and confounder influence. This study aimed to compare biomarker potential for different molecules and diseases. We leveraged summary statistics of five blood quantitative trait loci studies (N = 1980 to 22,609) and genome-wide association studies (N = 9725 to 500,199) from 14 different brain disorders, such as Schizophrenia (SCZ) and Alzheimer’s Disease (AD). We applied summary-based and two-sample Mendelian Randomization to estimate the associations between blood molecules and brain disorders. We identified 524 RNA, 807 methylation sites, 29 proteins, seven cytokines, and 22 metabolites having a significant association with at least one of 14 brain disorders. Simulation analyses indicated that a cross-omics combination of biomarkers had better performance for most disorders, and different disorders could associate with different omics. We identified an 11-methylation-site model for SCZ diagnosis (Area Under Curve, AUC = 0.74) by analyzing selected candidate markers in published datasets (total N = 6098). Moreover, we constructed an 18-methylation-sites model that could predict the prognosis of elders with mild cognitive impairment (hazard ratio = 2.32). We provided an association landscape between blood cross-omic biomarkers and 14 brain disorders as well as a suggestion guide for future clinical discovery and application.

## 1. Introduction

The diagnosis of chronic brain disorders at the present day is primarily dependent on clinical symptom assessments, which suffers from the drawback of subjectivity, symptom heterogeneity, and disease comorbidity [1]. To overcome these difficulties and aid early intervention of brain disorders, researchers have made considerable efforts to find objective diagnostic and predictive biomarkers [2]. Among all potential biomarkers, peripheral blood molecules, such as RNA [3], methylation site [4], and proteins [5] have gained specific attention due to the high feasibility and relatively low costs. So far, researchers have found a large number of potential blood biomarkers for the diagnosis of brain disorders [6]. 

However, the study and application of blood-based biomarkers has long suffered from the lack of reproducibility [6]. Overfitting is one of the main challenges: a transcriptome-wide analysis typically has more than 20,000 RNAs detected, whereas the sample size of test subjects is usually limited to no more than a few hundred due to the labor cost burden. The conflicts between feature number and the sample size are even exacerbated when the biomarkers’ discovery strategy of “multi-omics” is applied [7]. On the other hand, technical, and biological confounders, such as batch effect, immune state, lifestyle, and influence from diseases of different organs, dramatically impact the level of blood molecules [6], which further beclouds the true association between biomarkers and brain disorders. 

Theoretically, Mendelian Randomization (MR) [8] could properly overcome the challenge of overfitting and confounder effects (Figure S1). By using genetic variants that significantly impact the expression level of a blood molecule (so-called Quantitative Trait Loci, QTL) [9,10,11,12,13] as instruments, MR separates participants into high- and low-level groups. Next, MR compares the prevalence of a disorder between two groups to evaluate the association between the molecule and the disorder. Furthermore, in a 2-sample MR (2SMR) scenario, the QTL-molecule and QTL-disease associations can be estimated in two different Genome-Wide Association Studies (GWAS) (Figure S1) and then integrated to estimate the final molecule-disease association. Such analyses overcome overfitting by profoundly increasing the effective sample size (i.e., combining samples of two GWAS) and eliminating confounders’ impact since the genetic instruments are unlikely to be influenced by environmental factors [8]. Based on the framework of 2SMR, Zhu et al. [14] proposed a new MR method, Summary statistic-based Mendelian Randomization (SMR), which better addresses the MR analysis of high-throughput data. Both SMR and 2SMR can serve as the ideal tools for biomarker discovery.

The explosive growth of large-scale QTL studies demonstrated the significant heritability of blood levels of RNA [11], methylation [12], protein [13], cytokine [10], and metabolite [9], which provided the opportunity of applying 2SMR and SMR to discover their association with brain disorders. In the current study, we integrated these QTL data and GWAS summary statistics of 14 brain disorders (Figure S1) to achieve three goals: (1) comprehensively evaluate the potential of biomarkers for each omic and disorder; (2) validate some of the top candidates using publicly available high-throughput datasets; and (3) provide a biomarker potentiality landscape, which could serve as a reference for a future biomarker study.

## 2. Materials and Methods

### 2.1. Data Collection and Pre-Processing

We downloaded blood QTL [9,10,11,12,13] and disease GWAS summary statistics from the public domain, including SCZ [15], Bipolar Disorder (BP) [16], Major Depressive Disorder (MD) [17], Anorexia Nervosa (AN) [18], Attention deficit hyperactivity disorder (ADHD) [19], General Anxiety Disorder (ANX) [20], Tourette’s syndrome (TS) [21], Obsessive-Compulsive Disorder (OCD) [22], Autism Spectrum Disorder (ASD) [23], Alcohol Dependence (ALD) [24], Post-traumatic stress disorder (PTSD) [25], Alzheimer’s Disease (AD) [26], Parkinson’s Disease (PD) [27] and Nicotine Dependence (ND) [28]. We searched for all blood QTL and GWAS studies that (1) provided effect size and standard error summary statistics for genome-wide variants; (2) recruited only European populations; (3) for the QTL study, only involved healthy participants. For each omic and disorder, we chose the study with the largest sample size. Readers who wish to replicate the study should gain approval from the corresponding authors of these data. We applied uniform filtration, pre-processing, and Surrogate Variable Analysis [29] (SVA)-based confounder adjustment on all datasets (Supplementary Notes).

### 2.2. Summary-Based and 2-Sample Mendelian Randomization (SMR and 2SMR)

For the association between RNA, methylation, protein markers, and brain disorders, we applied multi-SNP-based SMR [30], which utilized all cis-QTLs (*p* < 5 × 10^−8^) within 1-MB window of the markers to estimate the association. For the association between metabolite, cytokine markers, and brain disorders, we applied 2SMR by R package TwoSampleMR [31] using SNP with *p* < 1 × 10^−5^ on the entire genome. For blood-based markers of cytokines and metabolites, their QTLs did not have spatial implications, i.e., no “cis” or “trans” QTLs of a cytokine or a metabolite. Therefore, instead of SMR, we applied classic 2SMR, which included genome-wide significant QTLs as instruments, regardless of their genomic positions. For each QTL *i* of a marker m, SMR and 2SMR first estimated the effect of m on a disease d ($\beta_{md\left(i\right)}$) by Wald ratio

$$\beta_{md\left(i\right)}=\frac{\beta_{id}}{\beta_{im}}$$

where $\beta_{id}$ denoted effect of *i* on *d* (i.e., GWAS effect size of *i*) and $\beta_{im}$ denoted effect of *i* on m (i.e., QTL effect size of *i*). The SE (and corresponding statistics z) for each QTL was estimated by the delta method

$$SE_{md\left(i\right)}=\frac{SE_{id}}{\beta_{im}}$$

SMR and 2SMR then applied different methods to integrate multiple QTL results into the final estimation and corresponding *p*-value (Supplementary Notes). *p*-value adjustment was conducted separately for each omic-disease combination. Inflation factor λ was calculated on a quantile-quantile plot for each omic disease combination. We defined λ as the slope of Chi-square regression of actual *p*-value on expected *p*-value [32].

Next, we applied the Heterogeneity In Dependent Instruments test (HEIDI) [14] to evaluate whether these associations were driven by the co-localization (i.e., molecule level and disease do not share the same causal SNP, but their causal SNPs were in a strong Linkage disequilibrium). If the association was driven by the same causal SNP instead of colocalized SNPs, the HEIDI test would be expected to return a *p*-value > 0.05, for which we denoted the marker as HEIDI(+), *p*-value > 0.05 would be denoted as HEIDI(−).

### 2.3. Simulation Analysis

To quantify the classification power of markers from each omics, we generated simulation data with the hypothesis that SMR-estimated β_md truly reflected reality, and with the consideration of estimation uncertainty and environmental influence. Specifically, for each omic-disease combination, we repeated the following procedure 1000 times to generate 1000 simulation datasets:

(1) For marker *m* (*m* = 1, 2,…, *n*) from omic o of disease d, we generated normal distribution $\ddot{\beta_{md}}\sim N\left(\beta_{md},\;SE_{md}\right)$, where $\beta_{md}$ and $SE_{md}$ were effect size and SE obtained from SMR or 2SMR. We then generated a random $\ddot{\beta_{md}}$ from the normal distribution, which formed an effect size vector $B_{od}={\left\{\ddot{\beta_{md}}\right\}}_{m=1,2,\ldots n}$.

(2) We then generated a random expression matrix $E_{10,000\times n}$ by generating *n* random vectors of length 10,000 from N(0,1). This was because all OR from GWAS or QTL analysis has been standardized, such that β_md corresponded to log odds of d per 1-SD increment of *m*. To account for environmental confounders, we added a random noise of N(0,0.01) on each vector.

(3) We calculated the odds of *d* as $ODD\left(d\right)={\left\{odd_{i}\right\}}_{i=1,2,\ldots 10,000}=E\times B_{od}$, and subsequently, the probability of *d* as $P\left(d\right)={\left\{p_{i}\right\}}_{i=1,2,\ldots 10,000}={\left\{\frac{1}{1+odd_{i}}\right\}}_{i=1,2,\ldots 10,000}$. For simplicity, the intercept term was set as zero, i.e., the number of cases of d is set to be identical to that of control.

(4) The label (case or control) for each of the 10,000 simulated samples was randomly decided, with the probability of being a case = *P*(*d*).

On each of the simulation datasets, we applied Logistic regression by rms R package, and recorded the AUC and R2. We took the median AUC and R2 across 1000 simulation for comparison. For cross-omic analysis, we pooled all markers of a disease, ranked them according to the absolute effect size, and generated simulation datasets of all these markers by the same procedure. In each simulation data, we sequentially applied Logistic regression on top 1, top2, …top *n* markers and recorded the AUC, R2, and AIC (by MASS R package). We calculated the median values across 1000 simulations, and chose the optimal model with the lowest median AIC. All the above simulation analysis was carried out separately for HEIDI(+) and HEIDI(−) markers.

### 2.4. Published Transcriptome and Methylome Data Analysis

For public transcriptome or methylome data, we extracted the value of HEIDI(+) and HEIDI(−) markers, applied Logistic regression, and recorded the AUC. the obtained AUC was compared to the corresponding simulation AUC (restricted to markers available in the real data). To compare the power of HEIDI(+) and HEIDI(−) markers, we ranked the HEIDI(−) markers according to their SMR *p*-value and chose top markers with the same number of HEIDI(+) markers. We applied Logistic regression on these two sets of markers of the same number and compared their AUC and log-likelihood. 

### 2.5. Diagnostic Model Construction

For SCZ methylation markers, we calculated the Spearman correlation coefficient ρ between each of the 1897 SMR-identified markers (both HEIDI(+) and HEIDI(−)) and diagnostic status, and retained only those with (1) ρ and SMR β of same direction; (2) |ρ| > 0.05. Then, we applied a Bayesian LASSO (bLASSO) regression by monomvn R package on the remaining 480 markers. All markers with median posterior coefficients not equal to zero were chosen as candidate marker. In the training set, we applied classical LASSO regression on the candidate markers. All remaining markers, together with their non-zero coefficients, constructed the final diagnostic model. We determined the optimal cut point using cutpointr R package by maximizing Youden’s Index. Finally, the coefficient as well as cut point of the identified model were fixed and applied to the validation set

### 2.6. Predictive Model Construction

We downloaded from ADNI repository all blood methylation data for which the diagnosis at sample collection was “MCI” (mild cognitive impairment), except those recovered from dementia status. ADNI project collected blood samples from elders at MCI status, and we used these methylation data at the beginning of the observation as potential predictors of future conversion risk. The sample information could be downloaded from ADNI repository after application approval. According to whether the participants converted to AD in the entire follow-up period recorded by ADNI, we classified samples in the training set as converter and non-converter. We first carried out Spearman correlation analysis and LASSO regression similar to the diagnostic model. Then, in the validation set, we applied this model to define high conversion risk and low risk group. The hazard ratio as well as its *p* value was calculated by univariate Cox regression using survival and survminer R package. Details of diagnostic and predictive model construction and be found in the Supplementary Notes.

## 3. Results

### 3.1. Identifying All Potential Blood-Based Biomarkers Associated with Brain Disorders

After data filtering (Supplementary Notes), we collected a set of valid QTLs for blood expression levels of 15,052 RNA, 89,910 methylation sites, 669 proteins, 41 cytokines, and 119 metabolites, on which we applied the MR Method. By restricting at the genome-wide significance threshold (*p* < 0.05/*n*, where *n* denoted the number of tested molecules of the corresponding omics), we identified 1386 blood-based molecular markers, including 524 RNAs, 807 methylation sites, 29 proteins, seven cytokines, and 19 metabolites, which had a significant association with brain disorders (Tables S1–S5). We then considered them as potential biomarkers of related diseases in the following analyses. 

Next, we applied the Heterogeneity In Dependent Instruments test (HEIDI) to evaluate whether these associations were driven by the co-localization (i.e., molecule level and disease do not share the same causal SNP, but their causal SNPs were in a strong Linkage disequilibrium). Among all potential biomarkers identified by SMR, we found that 82% of RNA, 80% of methylation, and 62% of proteins showed a HEIDI *p* < 0.05 (HEIDI(−)), suggesting that co-localization made a significant contribution to the identified association between peripheral molecules and diseases. Since we do not know whether or not co-localization would influence the biomarker predictivity, we analyzed HEIDI(+) (HEIDI *p* > 0.05) and HEIDI(−) markers separately in the following section to deal with the potential heterogeneity between them. 

### 3.2. RNA and Methylation Levels Showing Strong Associations with SCZ, PD, and AD

**Figure 1:**
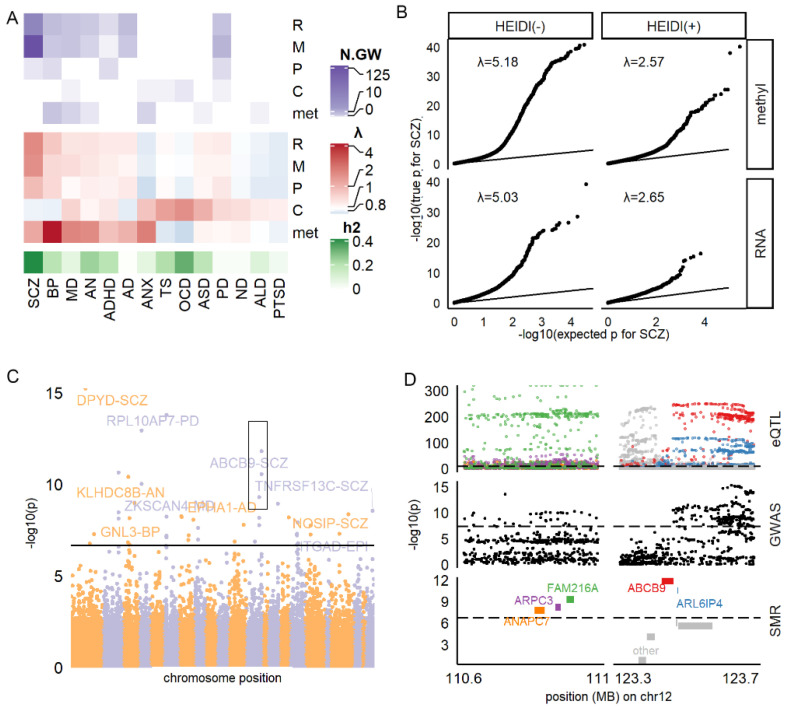
Summary of SMR results. (**A**) Number of HEIDI(+) molecules reaching genome-wide significance (N.GW; top panel) and inflation factor (middle panel) for each omic and disease. SNP-based heritability (h2) was estimated by Linkage Disequilibrium Score Regression. (**B**) QQ plot for SMR result of RNA-SCZ and methylation-SCZ association. (**C**) Manhattan plot for the association between RNA and all diseases. (**D**) Regional GWAS, QTL, and SMR statistics for SCZ in distal 12q region (black box in (**C**)) E: selected top molecules associated with multiple diseases. F: association between cytokines, metabolites, and diseases. R: RNA, m/methyl: methylation site. P: protein. C: cytokine. met: metabolite.

To identify the RNA, methylation, and protein biomarkers and their characteristics, we applied cis-QTL SMR and downstream enrichment analysis on their QTL. When restricted on candidate biomarkers with HEIDI *p* > 0.05 (HEIDI(+)), SCZ had the largest number of RNA (*n* = 52) and methylation (*n* = 126) biomarkers, followed by PD (*n* = 8 and *n* = 14), BP (*n* = 10 and *n* = 7), and AD (*n* = 10 and *n* = 6, respectively, see Figure 1A). These diseases also had a large number of HEIDI(−) markers, as shown in Figure S2. This association was not restricted to the top blood molecules with *p*-value passing the genome-wide significance threshold, as the general *p*-value distribution of all molecules was inflated compared to the null uniform distribution (inflation factor λ > 1; Figure 1A,B). Concordantly, λ was even higher for HEIDI(−) molecules for these disorders (Figure 1B and Figure S2). In contrast, TS, OCD, ND, ALD, and PTSD did not have any RNA or methylation candidate markers, and their λ was also lower than 1 (Figure 1A).

For the RNA-disorder association (Figure 1C), we found 95 HEIDI(+) markers and 429 HEIDI(−) markers reaching the genome-wide significance threshold (Table S1), which gathered on specific regions of the genome. Figure 1D showed an example of a distal 12q region, where five SMR-significant genes (ANAPC7, ARPC3, FAM216A, ABCB9, and ARL6IP4) had close spatial distribution in accordance with the regional QTL and GWAS loci distribution. However, RNA markers showed neither enrichments in brain-related biological functions (Supplementary Methods; adjusted *p*-value of Gene Ontology, GO, analysis >0.05) nor enrichment in genes preferentially expressed in the brain [33], or different brain cell types [34] (Fisher’s exact test *P*, *P*_fisher_ > 0.05; Table S6). We concluded that the identified RNA markers might better reflect global alteration rather than brain abnormality during brain disorders.

For the methylation-disorder association (Figure S3), we found 164 HEIDI(+) and 643 HEIDI(−) markers reaching the genome-wide significance threshold (Table S2). We observed that the methylation markers enriched in genomic regions of DNase I Hypersensitivity Site [35] (*P*_fisher_ = 9.10 × 10^−5^, Odds Ratio [OR] = 1.42), promotors (*P*_fisher_ = 1.99 × 10^−5^, OR = 1.51), prefrontal cortex H3k27ac [36] peaks (*P*_fisher_ = 1.87 × 10^−6^, OR = 1.49), and H3k36me3 [36] peaks (*P*_fisher_ = 2.68 × 10^−17^, OR = 1.88), and the chromHMM [37] state 5 (weak transcription) (*P*_fisher_ =1.23 × 10^−6^, OR = 1.57, Table S7). These methylation markers tagged 223 proxy genes by falling within the regions, including UTR (17%), gene body (53%), and 1500 base pairs around the transcript start site (30%) of the corresponding genes. Interestingly, these proxy genes enriched in neuronal functions, such as long-term synaptic potentiation (adjusted *P*_fisher_ = 0.002, Figure S3). They were also nominally enriched in genes preferentially expressed in the brain (*P*_fisher_ = 0.013, OR = 1.61, Table S6), but not in genes specifically expressed in specific brain cell types. These results suggested that methylation markers might better capture central nervous system abnormality in brain disorders.

We also analyzed protein biomarkers of brain disorders by SMR. We found 11 HEIDI(+) markers reaching significance threshold (Table S3), corresponding to eight different proteins, which included ESAM (PSMR for SCZ = 1.57 × 10^−7^), GPNMB (PSMR = 3.01 × 10^−6^ for PD), FLRT3 (PSMR for BP = 1.06 × 10^−5^), and MANBA (PSMR for ADHD = 4.62 × 10^−5^). However, due to limited numbers (*n* = 11), they did not show any functional enrichment.

### 3.3. Cytokines and Metabolites Exhibiting High Pleiotropy

**Figure 2:**
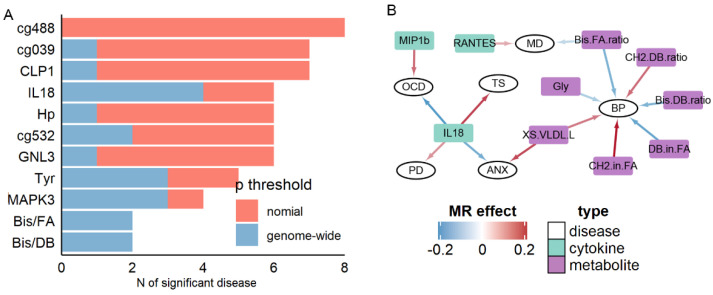
Summary of pleiotropic results. (**A**) Selected top molecules associated with multiple diseases. (**B**) Association between cytokines, metabolites, and diseases.

We next analyzed cytokines and metabolites that had an association with brain disorders. As shown in Figure 2 and Table S4, the blood level of IL18 was significantly associated with four disorders: TS (β = 0.19, *p* = 3.69 × 10^−7^), OCD (β = −0.19, *p* = 6.07 × 10^−5^), ANX (β = −0.15, *p* = 0.0002) and PD (β = 0.10, *p* = 0.0005). Similarly, another pleiotropic biomarker, the cytokine MIP1B, was also associated with two disorders (ASD: β = 0.07, *p* = 1.33 × 10^−6^; OCD: β = 0.15, *p* = 3.10 × 10^−6^). We also observed the pleiotropic association for metabolite markers, shown in Figure 2A and Table S5. In addition, the ratio of bis-allylic in fatty acid (Bis.FA.ratio) was observed to be significantly associated with two disorders: BP (β = −0.14, *p* = 6.20 × 10^−6^), MD (β = −0.06, *p* = 2.87 × 10^−5^), while multiple metabolites, such as Glycine and ratio of double bonds in fatty acid, were shown to be associated with BP (Figure 2B).

To further illustrate the extent of pleiotropic association between blood-based markers and brain disorders, we summarized the number of associated disorders for all markers (Figure 2A). As stated above, cytokines and metabolites markers exhibited higher pleiotropic associations at the genome-wide significance: IL18, Tyrosine, the bis-allylic ratio in fatty acid, and bis-allylic ratio in double bond were associated with at least three disorders at genome-wide significance. In contrast, for RNA, methylation, and protein markers, only the RNA expression levels of MAPK3 were significantly associated with three disorders (SCZ, PD, and AD). Only when the significance threshold was relaxed to nominal *p* < 0.05, seven methylation biomarkers, such as cg20670488 (Figure 2A), were found associating with eight different disorders. Therefore, in general, at the stringent threshold, cytokines, and metabolites markers exhibited pleiotropic association with brain disorders.

Additionally, we applied step-wise outlier removal and MR sensitivity analysis (Supplementary Notes) to test for causality. We found that the MR result was not explained by pleiotropy or systemic bias (Table S8 and Figure S4), indicating that the association between biomarkers and disorders was causal. Thus, although biomarkers’ discovery did not require them to be causal [5], our potential cytokine and metabolite biomarkers for brain disorders still provided evidence of causality and yielded insights into the disease mechanism.

### 3.4. Simulation Demonstrated the Advantage of Using Cross-Omics Biomarker Combinations

Having identified the potential biomarkers from each of the five omics, we sought to quantify their diagnostic power and compare them across diseases and omics. Thus, for each omic-disease combination, we generated 1000 simulation datasets based on the estimated effect size of each biomarker (Method) and calculated the Area Under Curve (AUC) and Nagelkerke pseudo-R square (R2) in each dataset. 

**Figure 3:**
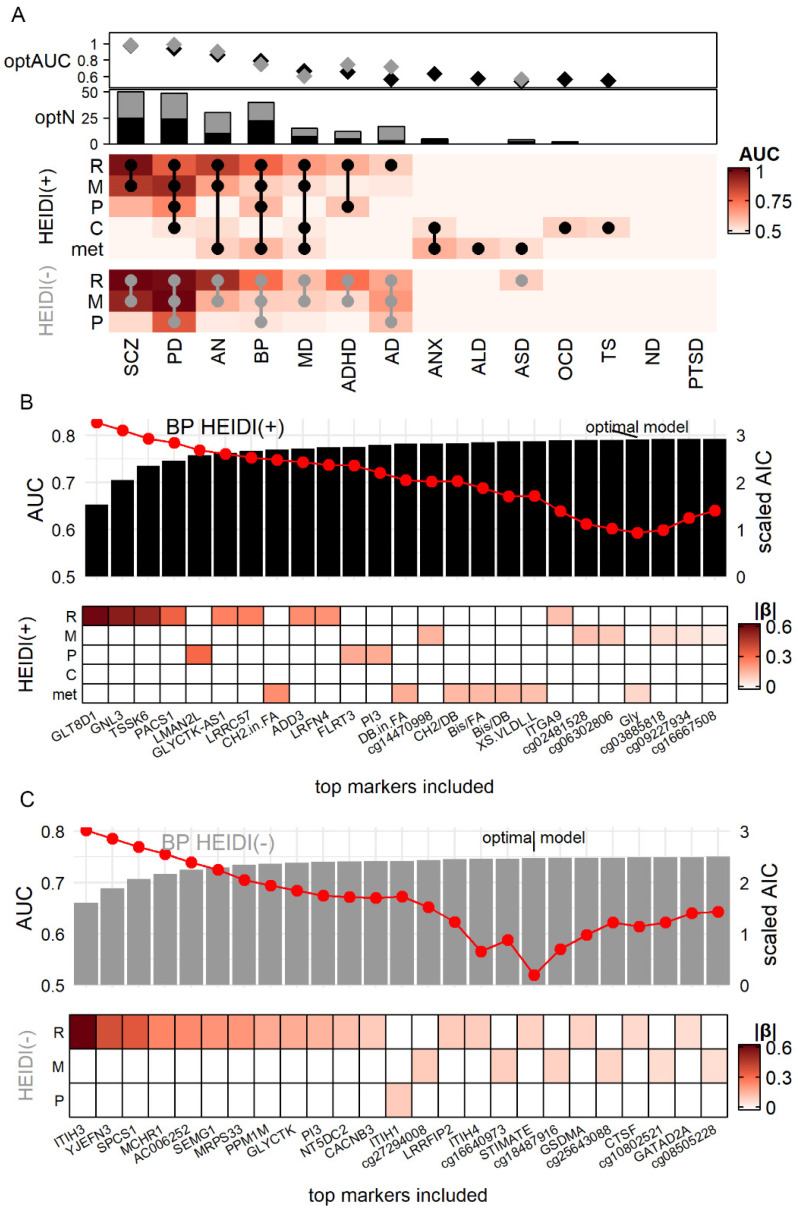
Comparison of biomarker efficiency among diseases and omics in simulation. (**A**) Each grid of the heatmap showed the Area Under Curve (AUC) of the model consisting of all markers from one omic for one disease. Dots on the heatmap showed the composition of the optimal model with the lowest Akaike information criterion (AIC). Diamonds (optAUC: AUC for optimal model) and bar plots (optN: number of markers in optimal model) showed the AUC and number of markers of the optimal models (black for HEIDI(+) model, grey for HEIDI(−) model). (**B**,**C**) example optimal model of BP. Red dots denoted AIC. R: RNA. M: methylation site. *p*: protein. (**C**) cytokine. met: metabolite. |β|: absolute value of βSMR.

As shown in Figure 3A, RNA and methylation markers of SCZ and PD had the largest classification power (AUC = 0.78 to 0.96 for HEIDI(+), 0.92 to 0.99 for HEIDI(−)), which were mainly due to a large number of markers (Figure 1A and Figure S2). Nonetheless, some omics had a strong statistical power despite the small number of markers, such as the protein markers of PD (HEIDI(+): 3 markers, AUC = 0.70, R2 = 0.17), RNA markers of AN (HEIDI(+): 5 markers, AUC = 0.85, R2 = 0.46). In sum, we observed that RNA markers generally had the largest classification power: the median AUC for RNA was 0.73, whereas none of the other omics had a median AUC > 0.65. For six diseases (SCZ, AN, BP, MD, ADHD, and AD; Figure 3A), RNA HEIDI(+) markers had a larger AUC than other omics. 

We further explored whether a combination of different omics would gain a better diagnostic power. Figure 2B,C showed the examples of BP biomarker combination, which showed association with four omics. We generated simulation data (Method) of all 26 BP markers and calculated the Akaike information criterion (AIC) to choose the optimal combination of markers. The combination with the lowest AIC had the best trade-off between marker numbers and diagnostic efficiency [38]. We found that the model of the top 22 markers had the lowest AIC (Table S9), which achieved the AUC of 0.79 and R2 of 0.33. This model included RNA markers such as GLT8D1, methylation markers such as cg14470998, protein markers such as LMAN2L, and metabolite markers such as CH2.in.FA (CH2 ratio in fatty acid), suggesting that combining markers from different omics might gain a better power of classifying BP patients from a healthy control. A similar analysis was also carried out for HEIDI(−) BP markers, where an 18-marker model consisted of three omics had the lowest AIC (Figure 3C).

Taking the results of all diseases together, we found that the optimal models of seven diseases, from SCZ to AD in Figure 3A, were cross-omics (round dots on Figure 3A denoted the composition of the optimal model). Of note, the optimal HEIDI(+) model of AN achieved AUC = 0.87 and R2 = 0.50 with only 10 multi-omics markers. This model consisted of large-effect markers IMPDH2 (βSMR = 1.48), CADM1 (βSMR = 0.49) (Figure S5). Another noteworthy disease was AD (Figure S6): its HEIDI(+) model had poor performance (three RNA markers, AUC = 0.57, R2 = 0.02), but the HEIDI(−) model (14 markers from three omics, AUC = 0.72, R2 = 0.19) yielded better performance. 

### 3.5. HEIDI(+) and HEIDI(−) Markers Having Comparable Power in Real-World Validation

We next sought to validate the classification power implicated by simulation analysis in the real-world data. We collected 12 cross-sectional blood RNA data [3,39,40,41,42,43,44,45,46,47,48,49] of seven diseases and 11 blood methylation data [4,50,51,52,53,54,55,56,57,58] of six diseases to evaluate the efficiency of RNA and methylation markers. We did not analyze protein, cytokine, and metabolite markers since limited public data is available. As shown in Figure S7, methylation markers of AD, BP, MD, and AN generally had higher AUC in real data than in simulation data, especially HEIDI(−) markers of AN (real AUC = 0.85, simulation AUC = 0.63). On the other hand, RNA and methylation markers of SCZ and PD tended to have lower AUC in the real data, suggesting that only a small proportion truly took effect among the large number of SMR-identified markers of SCZ and PD. 

We then investigated whether the power of HEIDI(−) markers was comparable to HEIDI(+) markers. We observed that HEIDI(+) and HEIDI(−) markers generally had similar AUC. Despite a few exceptions, including methylation markers of AN (HEIDI(+) AUC = 0.76, HEIDI(−) AUC = 0.65), the difference of AUC of HEIDI(+) and HEIDI(−) markers were generally smaller than 0.05. Concordantly, the Likelihood ratio and the number of significant variables of Logistic regression were also similar for HEIDI(+) and HEIDI(−) markers (Table S10), which suggested that their classification power and significance were similar. Therefore, we no longer distinguished HEIDI(+) and HEIDI(−) markers in the following section. 

### 3.6. Construction of Molecular Diagnostic Models for SCZ and AD with Notable Accuracy

**Figure 4:**
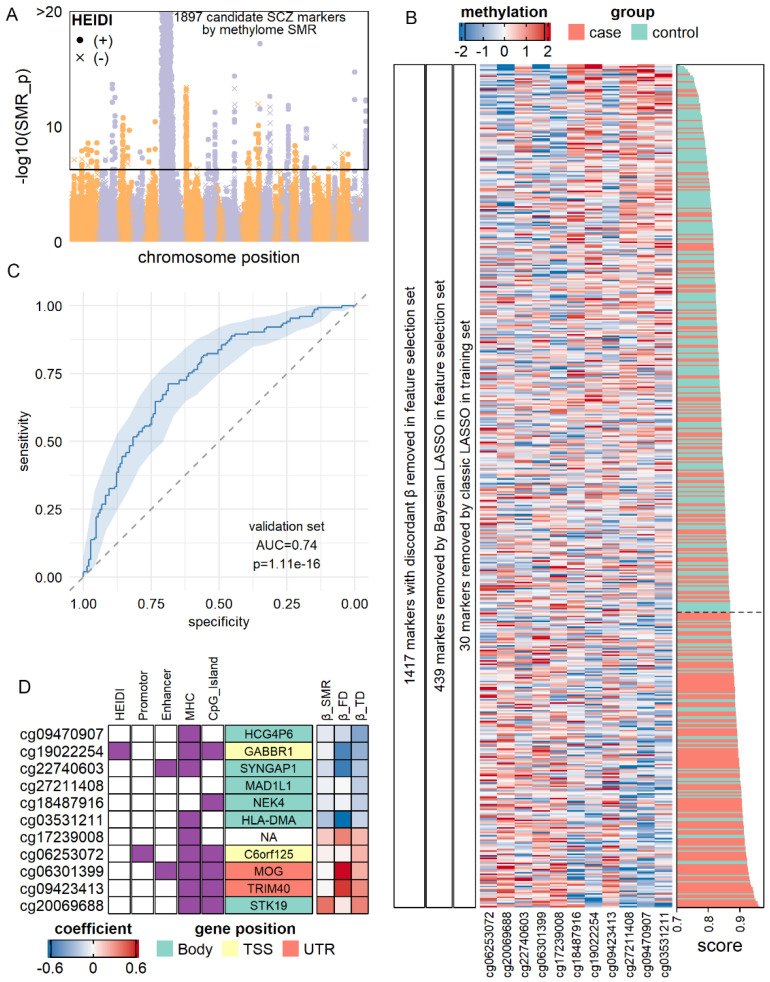
Validation of methylation markers of SCZ. (**A**) Manhattan plot for SCZ-methylation association by SMR. Each dot represents a methylation site. (**B**) LASSO model of SCZ diagnostic model by methylation markers in the training set (N = 547). Heatmap showed the expression levels of 11 markers after filtration and model construction in the training set. Score: diagnostic scores calculated by the sum of methylation levels weighted by the LASSO coefficient. A higher score corresponded to a higher probability of being an SCZ patient. (**C**) Receiver-Operation Curve for LASSO model in the validation set (N = 300). (**D**) Characteristics of methylation markers chosen by LASSO. Gene symbols denoted the proxy genes of each methylation marker, and their grid color denoted the position of the methylation markers on the proxy genes. Purple girds on column “HEIDI” indicated that the markers had *p* > 0.05 in HEIDI. β_FD_: coefficients in the feature selection set. β_TD_: coefficients in the training set.

Next, we sought to construct optimal models with a subset of top markers by validating the candidate biomarkers in public datasets. After general consideration of biomarker effect size and available sample size, we decided to analyze methylation markers of SCZ, PD, and AD, as well as RNA markers of BP and SCZ (Figures S8 and S9). For SCZ, we divided blood methylation datasets from Hannon et al. [59] into feature selection set (N = 675), training set (N = 547), and validation set (N = 300). Since the number of candidate methylation SCZ markers (N = 1897; Figure 4A) was extremely large, we applied Spearman correlation analysis and Bayesian LASSO (Method), which removed a total of 1856 markers without concordant coefficients in SMR and the feature selection set. Then, we applied classic LASSO on the remaining 41 candidates in the training set (N = 547) and obtained a linear classification model consisted of 11 methylation sites (Figure 4B) with AUC = 0.72 (95% CI of 0.67–0.76). Next, we fixed the coefficient of each predictor as well as the optimal cut point (obtained by maximizing Youden’s Index) [60] and applied the model to the validation set (N = 300). The model achieved AUC = 0.74 (95% CI of 0.69–0.80, Figure 4C) with accuracy of 0.70 (sensitivity = 0.71 and specificity = 0.69). The proxy genes of these markers included GABBR1, which encoded a subunit of gamma-aminobutyric acid receptor, SYNGAP1, which encoded a member of N-methyl-D-aspartate receptor complex, and MOG, which took part in oligodendrocyte myelination (Figure 4D). These results indicated that our 11-site model not only robustly classified SCZ patients from healthy control but also had a biological significance that could provide insight into SCZ pathology.

For AD, we downloaded the methylation dataset from ADNI [4] repository. All 74 SMR candidate markers (Figure S10A) were retained for analysis. In the training set (N = 600), we removed 45 out of 74 candidates due to discordant effect size compared with βSMR. We then applied LASSO regression on the remaining 29 candidates and obtained a model of 18 methylation sites (Figure S10B). This model had AUC = 0.79 (95% CI, 0.75–0.83), with diagnostic accuracy = 0.76 (sensitivity = 0.80 and specificity = 0.68). We then applied this model to the validation set (N = 321, Figure S10C) and observed diagnostic AUC = 0.73 (95% CI, 0.67–0.79) and accuracy = 0.70 (sensitivity = 0.73 and specificity = 0.64). These markers recurrently tagged lipoproteins, including, APOE, APOC1, and APOC2 (three times), and APOC4 (Figure S10D), in line with the popular notion that lipoprotein plays a vital role in the pathology of AD [58,61].

We also carried out a similar analysis for PD methylation markers as well as SCZ and BP RNA markers. For PD methylation markers (Figure S8), we obtained a three-site model with AUC = 0.65 (95% CI, 0.62–0.68) in the training set (N = 1200) and AUC = 0.66 (95% CI, 0.61–0.69) in the validation set (N = 689). For BP RNA markers (Figure S9), we found six genes (SPCS1, CTSF, ITGA9, ITIH4, PLAAT3, and PI3) out of 29 candidates in the training set (N = 360). This model achieved AUC = 0.71 (95% CI, 0.67–0.77) in training set and AUC = 0.63 (95% CI, 0.53–0.73) in the validation set (N = 120). For SCZ RNA markers, the identified model did not show significant classification power in the validation set.

### 3.7. SMR-Identified Methylation Markers Predicting the Risk of AD

As noted, our analysis considered all SMR-identified markers as diagnostic markers that reflected the current status of patients. However, predicting future disease prognosis is also an important task of biomarkers. Thus, we analyzed whether SMR-identified markers could serve as predictive biomarkers. Since a very limited number of the dataset had longitudinal records available, we only analyzed ADNI data of mild cognition impairment (MCI) elders and managed to predict their future conversion to AD by blood methylation data.

**Figure 5:**
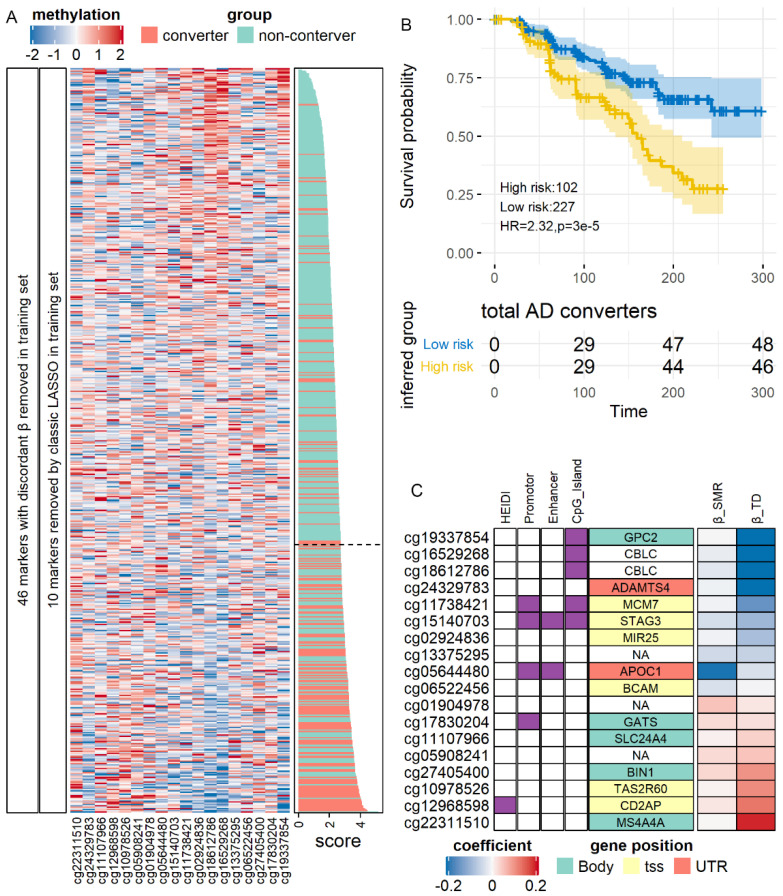
Validation of methylation predictive markers of AD. (**A**) Expression of markers after filtration and model construction in the training set. (**B**) Survival curve for conversion-to-AD in the test set. Time was shown in days. (**C**) Characteristics of the markers. HR: Hazard Ratio.

As shown in Figure 5A, we analyzed all 74 SMR-identified AD methylation candidates. In the training set (N = 600), LASSO regression returned an 18-site model which could distinguish converters from non-converters at the accuracy of 0.76 (sensitivity, 0.71; specificity, 0.77). The AUC in the training set was 0.79 (95% CI, 0.75–0.83). Using this model and its optimal cut point estimated in the training set, we classified the validation set into a high conversion risk group (N = 102) and a low-risk group (N = 227). In survival analysis (Figure 5B), we found that the high-risk group had a significantly lower interval of non-conversion survival (Hazard ratio = 2.32, *p* = 3.1 × 10^−5^). In the low-risk group, 74% (137) of the MCI patients did not convert to AD in the follow-up period of 300 days, whereas, as in the high-risk group, the median non-conversion survival times were 159 days. Similar to the diagnostic model (Figure S10D), these predictive markers’ proxy genes, including APOC1, GPC2, and SLC24A4 (Figure 5C). In sum, our results indicated that SMR-identified markers of AD could serve as both diagnostic and predictive markers.

## 4. Discussion

In the current study, we applied SMR and 2SMR on the QTL and GWAS statistics to evaluate the association between blood-based molecular markers and different brain disorders. We confirmed that blood levels of various multi-omics molecular markers had a significant association with brain disorders and may serve as both diagnostic and predictive biomarkers.

One of our major findings of the current study is that the peripheral molecules indeed carry information reflecting the central nervous system. The peripheral blood receives substance from all organs and tissues of the body, and its molecular composition is very different from the brain [62]. Traditional cross-sectional analysis has restricted power to decode the potential signals hidden within. In contrast, our MR-based approach, which enabled satisfactory statistical power and confounder-free estimation [8], demonstrated the existence of an association between peripheral signal and brain disorders. Another controversy is that, judging from the biological significance, MR association from colocalized QTL and GWAS SNP is of little interest [14]. However, we found that biomarkers identified by such association (HEIDI(−) markers) generally had comparable power with non-colocalized markers (HEIDI(+) markers). Thus, although co-localization introduced a challenge to GWAS and causality inference, their signals are still valuable for clinical biomarker study. After confirming the significant associations by MR, we further validated a subset of top candidate markers in the public data, and showed that this association was not masked by confounders and could be confidentially detected in the real world. Together, these results showed that the MR-identified candidate markers are highly promising for clinical application. 

Furthermore, our result highlighted the importance of multi-omics analysis. Current blood-based biomarker studies were predominantly restricted to transcriptome and methylome, while only a few studies focused on metabolites [63], proteins [5], and cytokines [64]. This discrepancy may be due to the different pace of technology advancement: next-generation sequencing and methylation array could comprehensively quantify genome-wide targets, whereas proteome and metabolome techniques could only cover a proportion of analytes. As a result, researchers might be prone to study transcriptome and methylome. However, our result showed that different brain diseases were associated with different omics, and not one omic could serve as biomarkers for more than half of the involved diseases. Thus, a reasonable solution might be a two-step design: selecting a few promising candidates from multiple omics and applied low-throughput validation in a large cohort. By validation of a few top molecules, we showed that potential markers identified MR in the current study could serve as promising candidates. However, these results could not rule out the impact of medications.

It should be noted that whether a biomarker is diagnostic or predictive is, theoretically, indistinguishable in MR analysis alone. The effect size β of MR is typically interpreted as: (1-SD) increment in biomarker levels changes the odds of disease to (eβ)-fold [65], similar to the coefficients of Logistic regression. Here, the “odds of disease” could be interpreted in the manners of both diagnosis and prediction: it could be “odds of being a patient now”, or “odds of becoming a patient in the future”. In our analysis of AD methylation markers in ADNI data [4], MR-identified markers fulfill the task of both diagnosis and prediction properly. This result suggested that it is reasonable to interpret the MR-identified markers as either diagnostic or predictive, and it is valuable to validate these markers in both manners.

However, there are still some limitations to the current study. MR requires that the tested molecule has a valid genetic basis. However, many of the blood-based molecules are not controlled by any genetic variants and could not be evaluated by MR. For these molecules, we could not draw conclusion about the lack of biomarker potentiality. Furthermore, there are minimal public data of blood proteome, cytokines, and metabolome available, which prevented us from validating candidate markers using them. The applicability of these candidates should be tested in future real-world studies.

## 5. Conclusions

In conclusion, our MR revealed that a blood biomarker study should focus on the most promising omics and molecules for the targeted disorders that we highlighted. We summarized all identified biomarkers and highlighted omics in the Supplementary Table S11. Our validation analysis using published data has shown that using this result as reference could profoundly improve the study efficiency and avoid overfitting. Future large multi-omic validation studies could prioritize the highlighted biomarkers in our study to achieve the final clinical models, which would reduce the risk of overfitting and confounder effects.

## Data Availability

All data generated in this study can be found in the supplementary tables.

## Supplementary Materials

The following are available online at https://www.mdpi.com/article/10.3390/jpm11121247/s1, Supplementary notes, including supplementary methods, supplementary Figures S1–S10 and supplementary Tables S1–S11. Supplementary Table S1–S5 All significant SMR result for transcriptome, methylome, proteome, cytokines, and metabolome. Supplementary Table S6 tissue- and cell type-specific expression. Supplementary Table S7 regional enrichment of methylation markers. Supplementary Table S8 Sensitivity tests for all heterogeneous MR result. Supplementary Table S9 Simulation analysis for BP. Supplementary Table S10 summary of results of real data analysis. Supplementary Table S11 Summary and comparison of blood multi-omic biomarkers. Supplementary Figure S1 Flowchart and data summary of the study. Supplementary Figure S2 summary of SMR HEIDI(−) results for each omic and disease. Supplementary Figure S3 methylation markers and its biological interpretation. Supplementary Figure S4 Step-wise outlier removal test for Bis.DB.ratio. Supplementary Figure S5 Simulation analysis of AN markers. Supplementary Figure S6 Simulation analysis of AD markers. Supplementary Figure S7 Comparison of HEIDI(+) and HEIDI(−) markers in real-world data. Supplementary Figure S8 Diagnostic model of PD by methylation markers. Supplementary Figure S9 Diagnostic model of BP by RNA markers. Supplementary Figure S10 Validation of methylation diagnostic markers of AD.
