# Supplementary material for: A Comprehensive Evaluation of Cross-Omics Blood-Based Biomarkers for Neuropsychiatric Disorders
Source: J Pers Med. 2021 Nov 24;11(12):1247. doi: 10.3390/jpm11121247 (PMC8703948; doi:10.3390/jpm11121247)
Supplement: Supplementary file 1 [file jpm-11-01247-s001.zip › 20210314_Supplementary materials.pdf]

Supplementary Notes for "A comprehensive evaluation of cross-omics blood-based biomarkers for brain disorders"

menu

Data collection and preprocessing ... 2
Phenotype definition ... 2
Summary-based Mendelian Randomization (SMR) ... 3
Two-sample Mendelian Randomization ... 3
Sensitivity analysis results for causality validation ... 4
Functional and regional enrichment of SMR-identified markers ... 5
Simulation analysis ... 5
Published transcriptome and methylome data analysis ... 6
Diagnostic model construction ... 7
Predictive model construction ... 7
Supplementary Table S1-S5 All significant SMR result for transcriptome, methylome, proteome, cytokines, and metabolome ... 8
Supplementary Table S6 tissue- and cell type-specific expression ... 8
Supplementary Table S7 regional enrichment of methylation markers ... 9
Supplementary Table S8 Sensitivity tests for all heterogeneous MR result ... 10
Supplementary Table S9 Simulation analysis for BP ... 10
Supplementary Table S10 summary of results of real data analysis ... 10
Supplementary Table S11 Summary and comparison of blood multi-omic biomarkers ... 11
Supplementary Figure S1 Flowchart and data summary of the study ... 12
Supplementary Figure S2 summary of SMR HEIDI(-) results for each omic and disease ... 12
Supplementary Figure S3 methylation markers and its biological interpretation ... 13
Supplementary Figure S4 Step-wise outlier removal test for Bis.DB.ratio ... 13
Supplementary Figure S5 Simulation analysis of AN markers ... 14
Supplementary Figure S6 Simulation analysis of AD markers ... 14
Supplementary Figure S7 Comparison of HEIDI(+) and HEIDI(-) markers in real-world data ... 15
Supplementary Figure S8 Diagnostic model of PD by methylation markers ... 16
Supplementary Figure S9 Diagnostic model of BP by RNA markers ... 17
Supplementary Figure S10 Validation of methylation diagnostic markers of AD. Similar to Figure 3, but for AD. ... 18
Reference ... 19

Data collection and preprocessing

We downloaded the cis eQTL data in SMR format from eQTLGEN website, which were derived from 26,609 participants. The methylation QTL data from McRea et al., which were derived from meta-analysis of 1980 participants, were downloaded from SMR website. The protein QTL data were obtained from Sun et al., which were derived from 3301 participants. We retained only QTL with $p<1\times10^{-5}$ and were within 1MB windows of the probes. Metabolite QTL and cytokine QTL were obtained from Kettunen et al. and Olli et al., and SNPs with $p<1\times10^{-5}$ on the entire genome were retained. We used the 1000 Genome Phase 3 (1000G) European cohort as the reference population for linkage calculation of SMR and 2SMR.

We downloaded the GWAS summary statistics of SCZ, BP, MD, AN, ADHD, ANX, TS, OCD, ASD, ALD, and PTSD from PGC website. Data for AD, PD, and ND were downloaded from the corresponding consortiums curated at traitDB[1] database. Since some of the GWAS did not provide allele frequency information, we uniformly removed all allele frequencies and replace them by frequency in 1000G. Only SNP with Minor Allele Frequency (MAF)>0.01 in the European population of 1000G were included. We log-transformed all OR to obtain zero-centered effect size for each SNP, and checked its direction to ensure that positive effect size was corresponded to increased disease risk. For SMR analysis, all data were transformed to .ma format required by SMR manually. In 2SMR analysis, for each molecule-disease pair, we extracted SNP with $p<1\times10^{-5}$ and were also recorded in the disease GWAS as instruments. They were then clumped to remove SNP with r2>0.001 with each other in 1000G. We harmonized the clumped instruments to remove incompatible alleles and make sure that the effect allele was uniform. These procedures were done by TwoSampleMR[2] R package separately for each molecule-disease pair.

We collected 13 cross-sectional blood RNA data of eight diseases and 11 blood methylation data of 6 diseases from GEO, ADNI repository, or directly from the corresponding authors. For diagnostic marker analysis, we removed all samples whose phenotypes were outside “case” and “control”. Detailed definition of phenotypes was provided in a case-by-case manner in below. For predictive marker analysis, only ADNI blood samples collected at “MCI” status were included. All array data were quantile-normalized and log-transformed, whereas RNA sequencing data were transformed into transcript-per-million (TPM) and log-transformed. Since covariates provided by each data were not uniform, we did not adjust for known covariates. Instead, we ran Surrogate Variable Analysis[3] (SVA) on each data and recorded all significant Surrogate variables (SVs). The normalized data were regressed against these SVs, and the scaled residual of regression was considered adjusted expression or methylation values. For multiple probes targeting the same genes, we retained the one with the largest average unadjusted values.

Phenotype definition

The methylation data of MD and BP was downloaded from Smith et al., which originally focused on the impact of childhood trauma. All participants of this study experienced childhood trauma to

some extent. We defined those participants with current usage of antidepressant treatment as MD cases, those with current usage of Mood stabilizers as BP cases, and those without any psychiatric medication as control.

For ADNI data, we removed all samples with recovery from any type of dementia to mild cognition impairment (MCI) or normal status. For the diagnostic model, the status label was defined as the diagnosis received at the closest time point of the blood collection. For the predictive model, the outcome was defined as all diagnoses received after the time point of blood collection.

Summary-based Mendelian Randomization (SMR)

For the association between RNA, methylation, protein markers, and neuropsychiatric disorders, we applied multi-SNP based SMR[4], which utilized all cis-QTLs ($p<5\times10^{-8}$) within 1-MB window of the markers to estimate the p-value of association. In brief, for each cis-QTL i of a marker m, SMR first estimated the effect of m on a disease d (β_{md}) by Wald ratio

$$\beta_{md(i)} = \frac{\beta_{id}}{\beta_{im}}$$

Where β_{id} denoted effect of i on d (i.e., GWAS effect size of i) and β_{im} denoted effect of i on m (i.e., QTL effect size of i). The SE (and corresponding statistics z_i) for each QTL was estimated by delta method

$$SE_{md(i)} = \frac{SE_{id}}{\beta_{im}}$$

. multi-SNP SMR then used the T statistic

$$T = \sum_i z_i$$

to generate the p-value of β_{md}, where the null distribution of T was estimated by LD correlation matrix. We then applied HEIDI test on all cis-QTL with $p<1\times10^{-5}$ to examine whether β_{md} was driven by different SNPs in strong LD. Details of HEIDI can be found at[4]. The p-value of SMR was adjusted by the Bonferroni method for each disease and each omic separately since the total number of available probes was slightly different for each disease.

Two-sample Mendelian Randomization

We applied 2SMR by TwoSampleMR R package using SNP with p<1×10-5 on the entire genome for the association between metabolite, cytokine markers, and neuropsychiatric disorders. Similar to SMR, we first estimated β_{md} for each SNP (so-called instrument) by Wald ratio, then meta-analyzed by three methods: Inverse-Variance Weighted (IVW), Weighted Median (WM), and MR Egger regression. The significance threshold was defined as: adjusted IVW $p<0.05$, WM $p<0.05$, β_{md} by IVW, WM and MR-Egger had the same direction. We adopted this threshold of significance to deal with the fact that we used a relative loose threshold for the inclusion of instruments ($p<1\times10^{-5}$), such that the influence of an invalid instrument should be taken into account. The β_{md} estimated by IVW was considered the primary result.

For all molecule-disease pairs, we used the Wald Ratio (i.e., dividing SNP-molecule effect by SNP-disease effect) to calculate the per-SNP estimation of the causal effect. We then meta-analyzed

all instruments by three methods:

1) Inverse Variance-Weighted (IVW) method, which calculated weighted mean (βIVW) of all per-SNP estimation. We directly applied exponential transformation on βIVW to generate the OR per 1-SD increment in exposure, as reported in the Result section.

2) Weighted Median (WM) estimation, which is the median of the weighted empirical distribution function of per-SNP estimation. WM can give precise estimation even when up to 50% of instruments are invalid.

3) Egger regression, a weighted linear regression of SNP-molecule against SNP-disease effect. By allowing a non-zero intercept, Egger regression controls the unbalanced pleiotropy and provides valid estimation even if all SNPs are invalid instruments, with the cost of low statistical power.

To analyze whether β_{md} reflected causal relation between marker m and disease d, we applied various sensitivity tests to rule out the possibility of horizontal pleiotropy[5], i.e., the causal SNP i impacts m and d via two distinct, horizontal mechanisms. P-value by any sensitivity test<0.05 indicated the existence of horizontal pleiotropy. These tests included:

1) the Cochran's Q test for IVW, a metric showing the extent to which all instruments deviated from the fitted line;

2) the Rucker's Q test for Egger regression, which also calculated the extent of instrument deviation, but also allowed for the non-zero intercept of the fitted line;

3) Egger intercept test: a significant non-zero Egger intercept indicated the existence of directional pleiotropy;

4) MR-PRESSO global test, which examined whether the observed residual sum of square (RSS_{obs}) exceed expectation by permutation.

If p values of at least one of the four tests <0.05, we further applied step-wise outlier removal test to get rid of the influence of pleiotropy. Specifically, we ranked all instruments in the descending order of RSSobs. We removed the top one instrument and repeated the three MR tests and four sensitivity tests on the remaining (n-1) instruments. If the p-value of at least one test was still smaller than 0.05, we repeated this procedure by removing top 2, top 3, … top (n-3) instruments, until all sensitivity tests had p-value >0.05 (leftmost black point in Figure S4). The β_{md} results at this point were denoted as the pleiotropy-free result.

Sensitivity analysis results for causality validation

As shown in Table S8, six out of seven cytokine-disorder associations, as well as 13 out of 22 metabolite-disorder associations, had p>0.05 in the four heterogeneity tests, indicating no heterogeneity. Thus, the association among them credibly reflected causality. For ten associations that failed one or more of the four heterogeneity tests, removing 3.5% to 31.9% of top outlier SNPs could yield a homogenous result (Table S8). As a typical example, the association between Bis.DB.ratio and BP (Figure S4) had Cochran p=0.02 and Rucker p=0.03 when all 47 instrument SNPs were included in the MR analysis. We then applied MR-PRESSO[6] to find potential outliers from the 47 SNPs and sequentially removed top1, top2, … top 44 outliers. When we removed 15 outliers (Figure S4), the remaining SNPs gave homogenous (i.e., p-value for four sensitivity test>0.05, Table S8) and significant MR result (β_{IVW}=-0.14, p=1.93×10^{-5}). Taken together, our 2SMR analysis not only found potential cytokine and metabolite biomarkers for neuropsychiatric disorders, but also found causality between

them, which yielded insights into the disease mechanism.

Functional and regional enrichment of SMR-identified markers

A list of genes preferentially expressed in the brain was downloaded from Genovese et al. [7]. To obtain the list of genes preferentially expressed in different brain cell types, we downloaded the single-cell data of Saunders et al. [8] and applied Expression Weighted Cell Type Enrichment[9] (EWCE) on it. Specifically, we applied "generate.celltype.data" function from EWCE R package to generate a gene×cell type specificity matrix, and we defined genes with top 20% specificity score of each major cell type as the preferentially expressed genes for this cell type. We tested whether RNA markers or proxy genes of methylation markers enriched in these gene lists by one-sided hypergeometric test, with background defined as all protein-coding genes. As for functional enrichment, we applied GO-BP analysis by clusterProfiler[10] R package, with a background set as all genes with GO-BP annotation and the p-value adjusted by FDR method.

For methylation site enrichment analysis, we obtained genomic annotations from three sources:

1) The following annotations were obtained from Illumina 450k documents: FANTOM methylation regions associated with promotor[11]; ENCODE enhancer[12]; DNase I Hypersensitivity Site[12]; CpG Island or shore; first exon, UTR, gene body, or 1500 bp around TSS of a gene.

2) The following annotations were obtained from the Roadmap project[13]: H3K9me3, H3K4me3, H3K27me3, H3K36me3, H3K9ac, H3K27ac, and H3K4me1 peaks of dorsal lateral prefrontal cortex.

3) The annotation of 15 core chromatin states was obtained from Ernst et al. [14].

We tested whether significant methylation markers were significantly enriched in these annotations by hypergeometric test, with background defined as all methylation sites with at least one strong ($p<5\times10^{-8}$) cis-QTL.

Simulation analysis

To quantify the classification power of markers from each omics, we generated simulation data with the hypothesis that SMR-estimated β_{md} truly reflected reality, and with the consideration of estimation uncertainty and environmental influence. Specifically, for each omic-disease combination, we repeated the following procedure 1,000 times to generate 1,000 simulation datasets:

1) For marker m (m=1,2…n) from omic o of disease d, we generated normal distribution $\ddot{\beta}_{md} \sim N(\beta_{md}, SE_{md})$, where β_{md} and SE_{md} were effect size and SE obtained from SMR or 2SMR. We then generated a random $\ddot{\beta}_{md}$ from the normal distribution, which formed an effect size vector $B_{od} = \{\ddot{\beta}_{md}\}_{m=1,2,\dots n}$.

2) We then generated a random expression matrix $E_{10,000\times n}$ by generating n random vectors of length 10,000 from $N(0,1)$. This was because that all OR from GWAS or QTL analysis has been standardized, such that β_{md} corresponded to log odds of d per 1-SD increment of m. To

account for environmental confounders, we added a random noise of $N(0, 0.01)$ on each vector.
3) We calculated the odds of d as $ODD(d) = \{odd_i\}_{i=1,2,\ldots 10,000} = E \times B_{od}$, and subsequently, the probability of d as $P(d) = \{p_i\}_{i=1,2,\ldots 10,000} = \left\{\frac{1}{1+odd_i}\right\}_{i=1,2,\ldots 10,000}$. For simplicity, the intercept term was set as zero, i.e., the number of cases of d is set to be identical to that of control.
4) The label (case or control) for each of the 10,000 simulated samples was randomly decided , with the probability of being a case $=P(d)$.

On each of the simulation datasets, we applied Logistic regression by rms R package, and recorded the AUC and R^2. We took the median AUC and R^2 across 1,000 simulation for comparison as in Figure 2.

For cross-omic analysis, we pooled all markers of a disease, ranked them according to the absolute effect size, and generated simulation datasets of all these markers by the same procedure. In each simulation data, we sequentially applied Logistic regression on top 1, top2, …top n markers and recorded the AUC, R^2, and AIC (by MASS R package). We calculated the median values across 1,000 simulations, and chose the optimal model with the lowest median AIC. All the above simulation analysis was done separately for HEIDI(+) and HEIDI(-) markers.

Published transcriptome and methylome data analysis

The public transcriptome or methylome data, as mentioned above, were first adjusted and scaled. We extracted the value of HEIDI(+) and HEIDI(-) markers, applied Logistic regression, and recorded the AUC. If more than one dataset were available for one disease, they were analyzed and recorded separately. The obtained AUC was compared to the corresponding simulation AUC (restricted to markers available in the real data).

To compare the power of HEIDI(+) and HEIDI(-) markers, we ranked the HEIDI(-) markers according to their SMR p-value and chose top markers with the same number of HEIDI(+) markers. We applied Logistic regression on these two sets of markers of the same number and compared their AUC, log-likelihood, and the number of markers with Wald test $p<0.05$ (and adjusted $p<0.05$).

We collected 12 cross-sectional blood RNA data[15, 16, 25, 26, 17–24] of seven diseases and 11 blood methylation data[27–35] of 6 diseases to evaluate the efficiency of RNA and methylation markers. We did not analyze protein, cytokine, and metabolite markers since limited public data is available. As shown in Figure S7, methylation markers of AD, BP, MD, and AN generally had higher AUC in real data than in simulation data, especially HEIDI(-) markers of AN (real AUC=0.85, simulation AUC=0.63). On the other hand, RNA and methylation markers of SCZ and PD tended to have lower AUC in the real data, suggesting that only a small proportion truly took effect among the large number of SMR-identified markers of SCZ and PD.

We then investigated whether the power of HEIDI(-) markers was comparable to HEIDI(+) markers. We observed that HEIDI(+) and HEIDI(-) markers generally had similar AUC. Despite a few exceptions like methylation markers of AN (HEIDI(+) AUC=0.76, HEIDI(-)

AUC=0.65), the difference of AUC of HEIDI(+) and HEIDI(-) markers were generally smaller than 0.05. Concordantly, the Likelihood ratio and the number of significant variables of Logistic regression were also similar for HEIDI(+) and HEIDI(-) markers (Table S10), which suggested that their classification power and significance were similar.

Diagnostic model construction

For SCZ methylation markers, we used the smaller data set (N=675) from Hannon et al. [35] as a feature selection set. Specifically, we calculated the Spearman correlation coefficient ρ between each of the 1897 SMR-identified markers (both HEIDI(+) and HEIDI(-)) and diagnostic status, and retained only those with 1) ρ and SMR β of the same direction; 2) $|\rho|>0.05$. Then, we applied a Bayesian LASSO (bLASSO) regression by "monomvn" R package on the remaining 480 markers. "bLASSO" took SMR β as prior coefficients for each marker, then applied a Markov chain Monte Carlo (MCMC) with chain length=1000 to generate posterior βdistribution. The initial LASSO penalty parameter was set at $\lambda^2=0.01$, and was not fixed along MCMC. All other parameters were set at the default of blasso() function. All markers with median posterior coefficients not equal to zero were chosen as candidate markers. In the training set (N=547, a subset from a larger dataset of Hannon et al. [35]), we applied classical LASSO regression on the candidate markers and estimated the coefficient with λ equal to minimum λ plus 1 SE. All remaining markers, together with their non-zero coefficients, constructed the final diagnostic model. Using this model, we calculated the diagnostic score for each sample and determined the optimal cut point using "cutpointr" R package by maximizing the Youden's index. The 95% confidence interval (CI) for ROC was evaluated by bootstrap using "ci.auc()" function, and the p-value of AUC was estimated by the z score method from the 95% CI. Finally, the coefficient, as well as cut point of the identified model, were fixed and applied to the validation set, and the AUC and accuracy were calculated similarly.

For the AD and PD methylation model as well as the BP RNA model, the number of SMR-identified markers was much lower. Thus, we did not use a feature selection set to pre-filter the candidate marker. Instead, we directly calculated Spearman ρ in the training set and removed discordant markers, as described above.

Predictive model construction

We downloaded from the ADNI repository[27] all blood methylation data for which the diagnosis at sample collection was "MCI", except those recovered from dementia status. Data were randomly separated into training (N=600) and validation (N=356) sets. According to whether the participants converted to AD in the entire follow-up period recorded by ADNI, we classified samples in the training set as converter and non-converter. We first carried out Spearman correlation analysis and LASSO regression similar to the diagnostic model and obtained a model that could distinguish converters from non-converters. Then, we applied this model to define high conversion risk and low-risk groups in the validation set. Hazard ratio and its p-value were calculated by univariate Cox regression using survival and "survminer" R

package. The endpoint of each sample was either conversion or cessation (last follow-up record).

Supplementary Table S1-S5 All significant SMR result for transcriptome, methylome, proteome, cytokines, and metabolome

Provided as a separate excel file.

Supplementary Table S6 tissue- and cell type-specific expression

term	RNA		methylation site	
	p	OR	p	OR
**Brain preferentially expressed**	**0.10**	**1.22**	**0.01**	**1.59**
**ASTROCYTE**	**0.54**	**0.99**	**0.13**	**1.29**
**CHOROID_PLEXUS**	**1.00**	**0.00**	**1.00**	**0.00**
**ENDOTHELIAL**	**1.00**	**0.19**	**1.00**	**0.00**
**EPENDYMAL**	**1.00**	**0.00**	**1.00**	**0.00**
**MACROPHAGE**	**0.34**	**1.09**	**0.42**	**1.09**
**MICROGLIA**	**1.00**	**0.00**	**1.00**	**0.00**
**MITOTIC**	**1.00**	**0.00**	**1.00**	**0.00**
**MURAL**	**0.37**	**1.07**	**0.22**	**1.22**
**NEUROGENESIS**	**1.00**	**0.00**	**1.00**	**0.00**
**NEURON**	**1.00**	**0.00**	**1.00**	**0.00**
**OLIGODENDROCYTE**	**1.00**	**0.00**	**1.00**	**0.00**
**POLYDENDROCYTE**	**1.00**	**0.00**	**1.00**	**0.00**

P and Odds Ratio are calculated by two-sided Fisher Exact test.

Supplementary Table S7 regional enrichment of methylation markers

term	All		HEIDI(+)		HEIDI(-)	
	p	OR	p	OR	p	OR
phantom	8.04E-02	1.35E+00	6.73E-01	8.72E-01	5.20E-02	1.46E+00
enhancer	8.04E-02	1.35E+00	6.73E-01	8.72E-01	5.20E-02	1.46E+00
DHS	9.10E-05	1.42E+00	3.45E-02	1.46E+00	2.59E-04	1.44E+00
Island	6.58E-01	9.68E-01	7.63E-01	8.85E-01	5.91E-01	9.82E-01
Shore	1.70E-02	1.17E+00	1.52E-03	1.61E+00	2.32E-01	1.07E+00
geneA	9.96E-02	1.62E+00	4.78E-02	3.01E+00	3.77E-01	1.21E+00
promoterA	1.99E-05	1.51E+00	2.69E-03	1.80E+00	1.37E-04	1.51E+00
fExon	8.72E-03	1.60E+00	1.77E-01	1.55E+00	2.01E-02	1.58E+00
UTR	6.51E-02	1.19E+00	5.46E-01	9.94E-01	7.35E-02	1.21E+00
body	3.32E-04	1.30E+00	5.15E-05	1.87E+00	6.13E-02	1.14E+00
TSS	4.16E-01	1.02E+00	8.16E-01	8.53E-01	1.27E-01	1.12E+00
h3k27ac	1.87E-06	1.49E+00	1.34E-02	1.53E+00	7.91E-06	1.52E+00
h3k27me3	1.00E+00	6.14E-01	1.00E+00	4.95E-01	1.00E+00	6.48E-01
h3k36me3	2.68E-17	1.88E+00	1.06E-08	2.46E+00	3.77E-12	1.78E+00
h3k4me1	4.48E-03	1.24E+00	6.91E-02	1.32E+00	1.88E-02	1.21E+00
h3k4me3	8.53E-04	1.28E+00	4.70E-02	1.34E+00	3.33E-03	1.27E+00
h3k9me3	9.53E-01	8.63E-01	9.69E-01	7.00E-01	8.33E-01	9.12E-01
1_TssA	2.10E-03	1.29E+00	1.76E-01	1.21E+00	1.32E-03	1.35E+00
13_ReprPC	9.92E-01	6.53E-01	9.95E-01	3.17E-01	9.48E-01	7.38E-01
2_TssAFlnk	2.40E-02	1.29E+00	9.61E-02	1.44E+00	4.82E-02	1.28E+00
11_BivFlnk	9.51E-01	5.11E-01	1.00E+00	0.00E+00	8.65E-01	6.45E-01
15_Quies	1.00E+00	6.38E-01	9.89E-01	6.30E-01	1.00E+00	6.38E-01
7_Enh	4.40E-01	1.03E+00	1.63E-01	1.32E+00	7.04E-01	9.30E-01
10_TssBiv	9.22E-01	7.12E-01	9.86E-01	2.31E-01	7.75E-01	8.35E-01
14_ReprPCWk	1.00E+00	6.09E-01	9.70E-01	6.27E-01	1.00E+00	5.82E-01
4_Tx	6.06E-02	1.32E+00	4.78E-01	1.08E+00	4.01E-02	1.41E+00
5_TxWk	1.23E-06	1.57E+00	4.34E-04	1.95E+00	2.80E-04	1.46E+00
6_EnhG	2.12E-02	1.89E+00	8.07E-01	6.09E-01	8.16E-03	2.21E+00
12_EnhBiv	9.18E-01	5.99E-01	5.38E-01	1.11E+00	9.61E-01	4.51E-01
9_Het	9.30E-01	3.75E-01	1.00E+00	0.00E+00	8.79E-01	4.73E-01
3_TxFlnk	2.46E-01	1.77E+00	1.00E+00	0.00E+00	1.57E-01	2.23E+00
8_ZNF/Rpts	1.00E+00	0.00E+00	1.00E+00	0.00E+00	1.00E+00	0.00E+00

Supplementary Table S8 Sensitivity tests for all heterogeneous MR result

Provided as a separate excel file.

Supplementary Table S9 Simulation analysis for BP

LR	N.marker	AUC	R2	AIC
744.783	1	0.652733	0.095697	13136.52
1350.438	2	0.70446	0.168436	12538.95
1791.641	3	0.735199	0.218714	12106.96
1962.947	4	0.745699	0.23765	11945.14
2178.82	5	0.75768	0.261047	11738.35
2261.588	6	0.762734	0.269893	11664.13
2338.397	7	0.766577	0.278021	11597.61
2385.576	8	0.769458	0.282991	11559.02
2424.841	9	0.771612	0.287099	11530.12
2468.25	10	0.773849	0.291656	11494.8
2484.299	11	0.774747	0.293307	11487.69
2562.408	12	0.778719	0.301395	11420.08
2620.169	13	0.781526	0.307355	11370.93
2635.585	14	0.78232	0.30892	11364.45
2644.516	15	0.782872	0.309832	11365.69
2682.738	16	0.784786	0.313736	11336.31
2717.489	17	0.786523	0.317281	11310.88
2725.784	18	0.786894	0.318126	11311.4
2761.764	19	0.788713	0.321763	11284.76
2781.055	20	0.789447	0.323731	11273.02
2794.366	21	0.790185	0.325066	11270.41
2805.592	22	0.790728	0.326186	11268.41
2812.766	23	0.791265	0.326924	11269.66
2814.079	24	0.791315	0.327053	11277.46
2816.763	25	0.791299	0.327319	11284.88

They corresponded to Figure 2B. For the ease of visualization, AIC was log-transformed and scaled in Figure 2B.

Supplementary Table S10 summary of results of real data analysis

Provided as a separate excel file

Supplementary Table S11 Summary and comparison of blood multi-omic biomarkers

Provided as a separate excel file

Supplementary Figure S1 Flowchart and data summary of the study

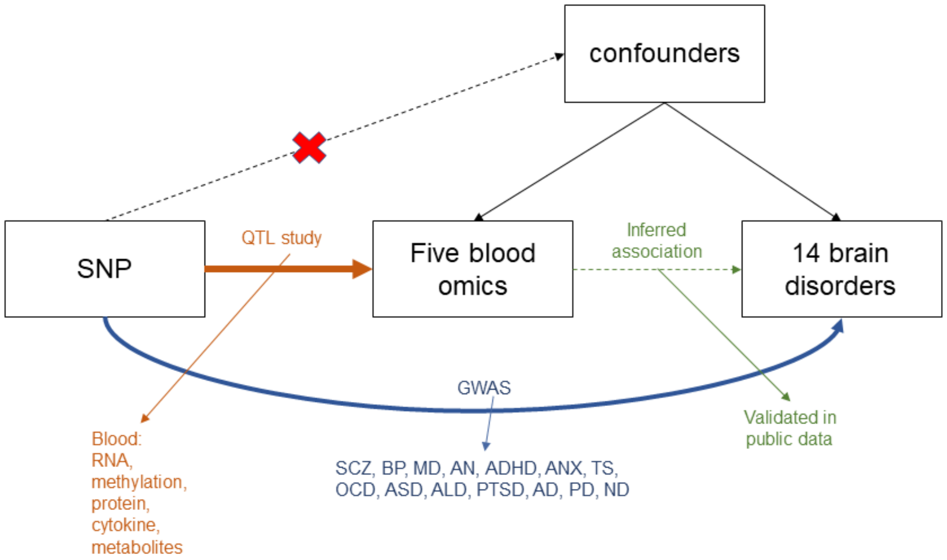

Supplementary Figure S2 summary of SMR HEIDI(-) results for each omic and disease

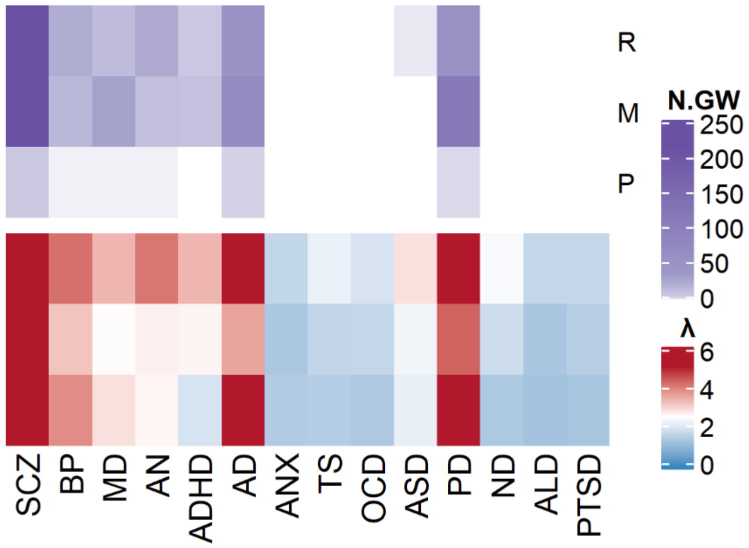

They corresponded to Figure 1A.

Supplementary Figure S3 methylation markers and its biological interpretation

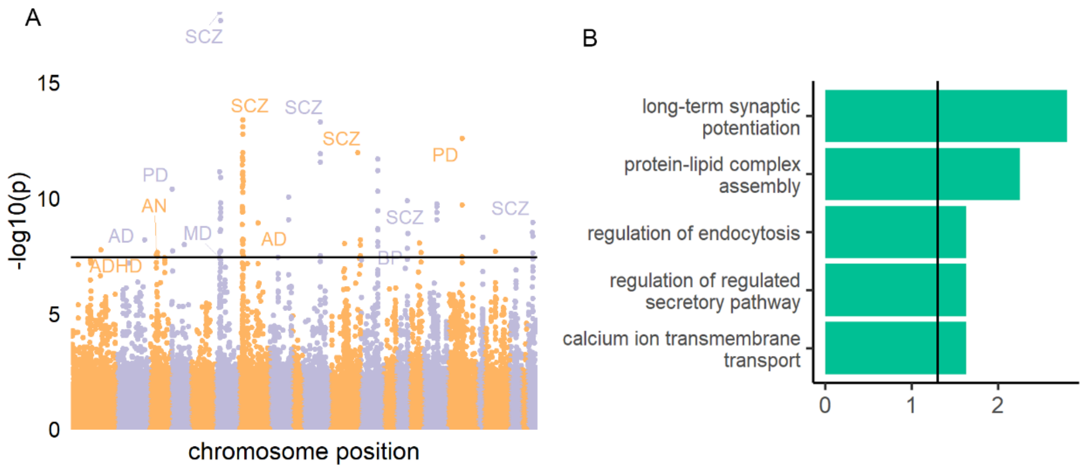

A: Manhattan plot of SMR methylation analysis. For the ease of visualization, we randomly removed 80% of points with p>0.01 as well as points within the MHC region. B: GO-BP analysis of proxy genes of SMR-identified methylation markers.

Supplementary Figure S4 Step-wise outlier removal test for Bis.DB.ratio

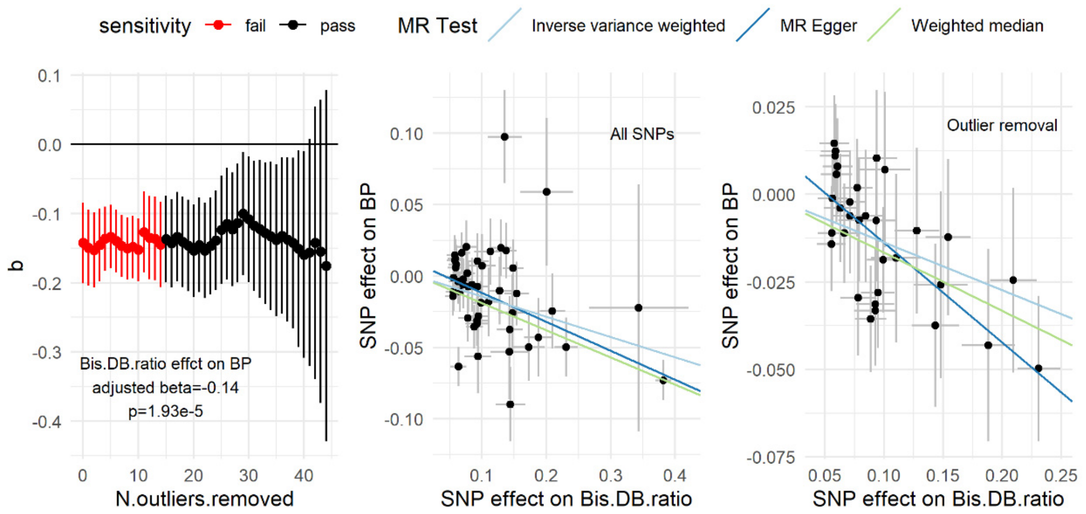

A: Each dot and its error bar showed IVW result after removing top outliers (x-axis). If the four sensitivity tests all had p>0.05, the dot was colored black. B: scatter plot of MR analysis with all instruments. C: scatter plot of MR analysis after removing outliers (corresponded to the first black dot in A).

Supplementary Figure S5 Simulation analysis of AN markers

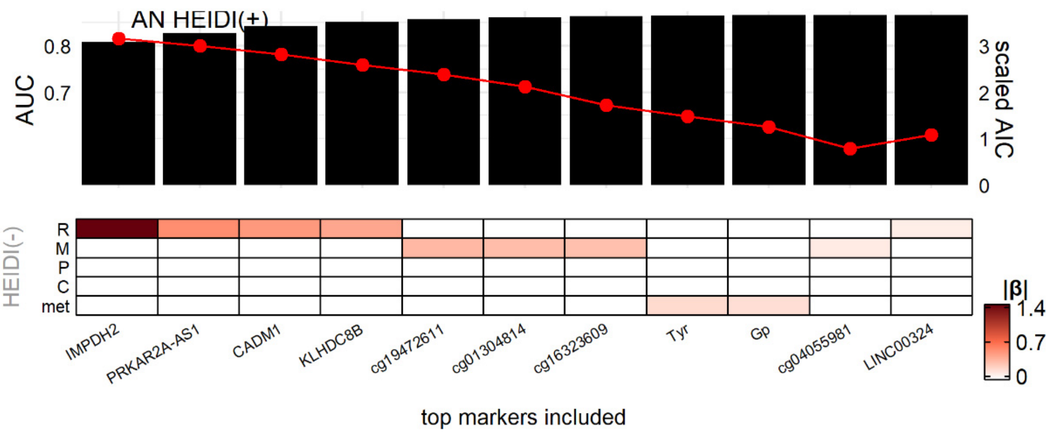

Similar to Figure 2B, but for AN.

Supplementary Figure S6 Simulation analysis of AD markers

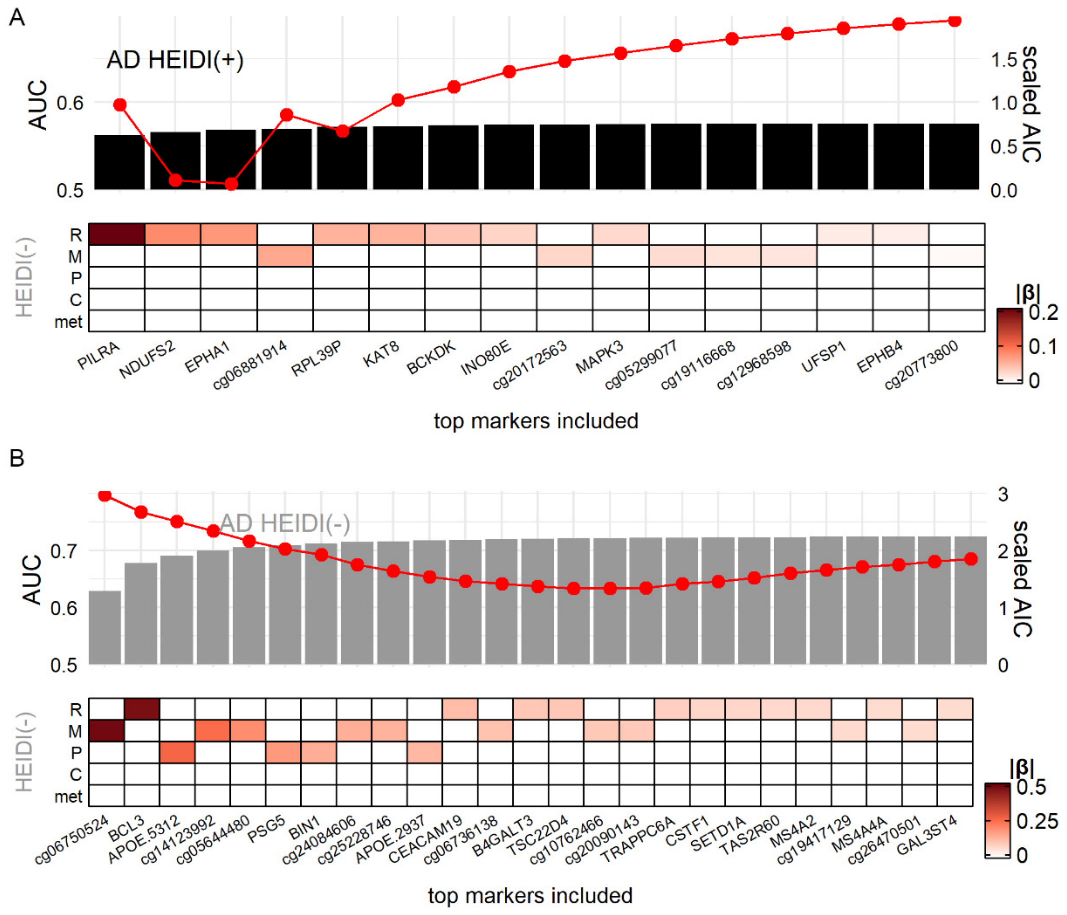

Similar to Figure 2B and C, but for AD.

Supplementary Figure S7 Comparison of HEIDI(+) and HEIDI(-) markers in real-world data

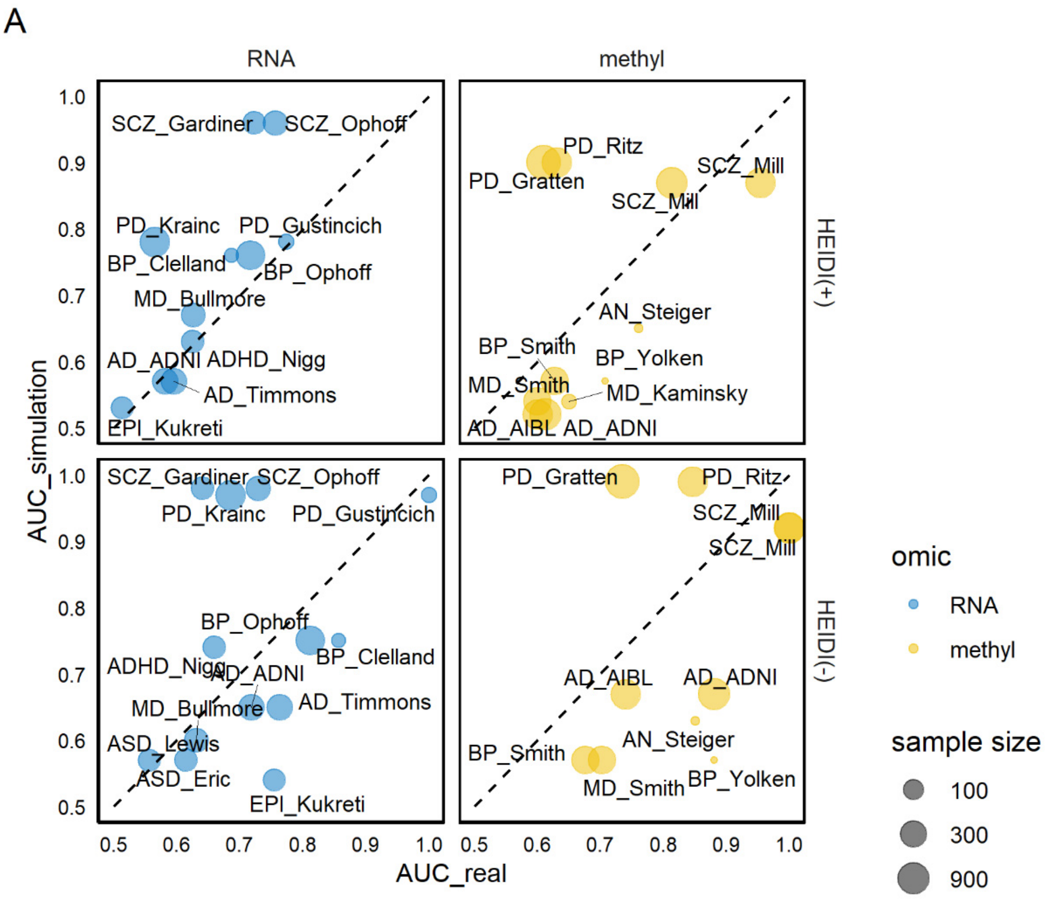

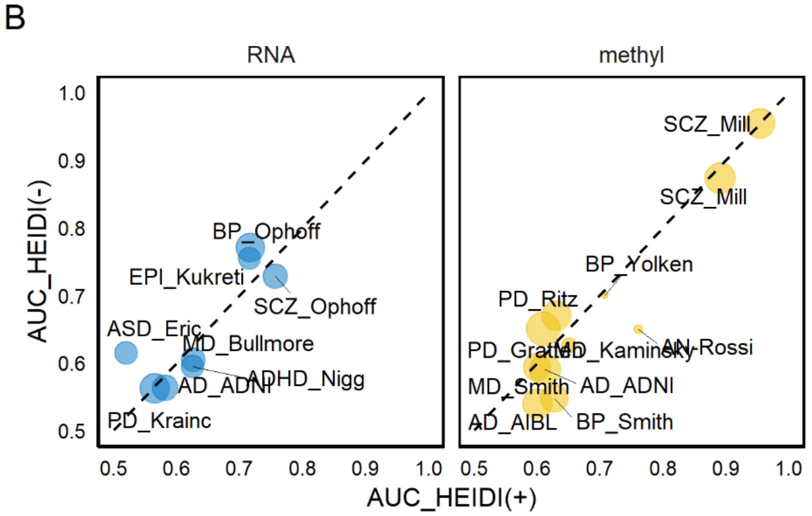

Supplementary Figure S8 Diagnostic model of PD by methylation markers

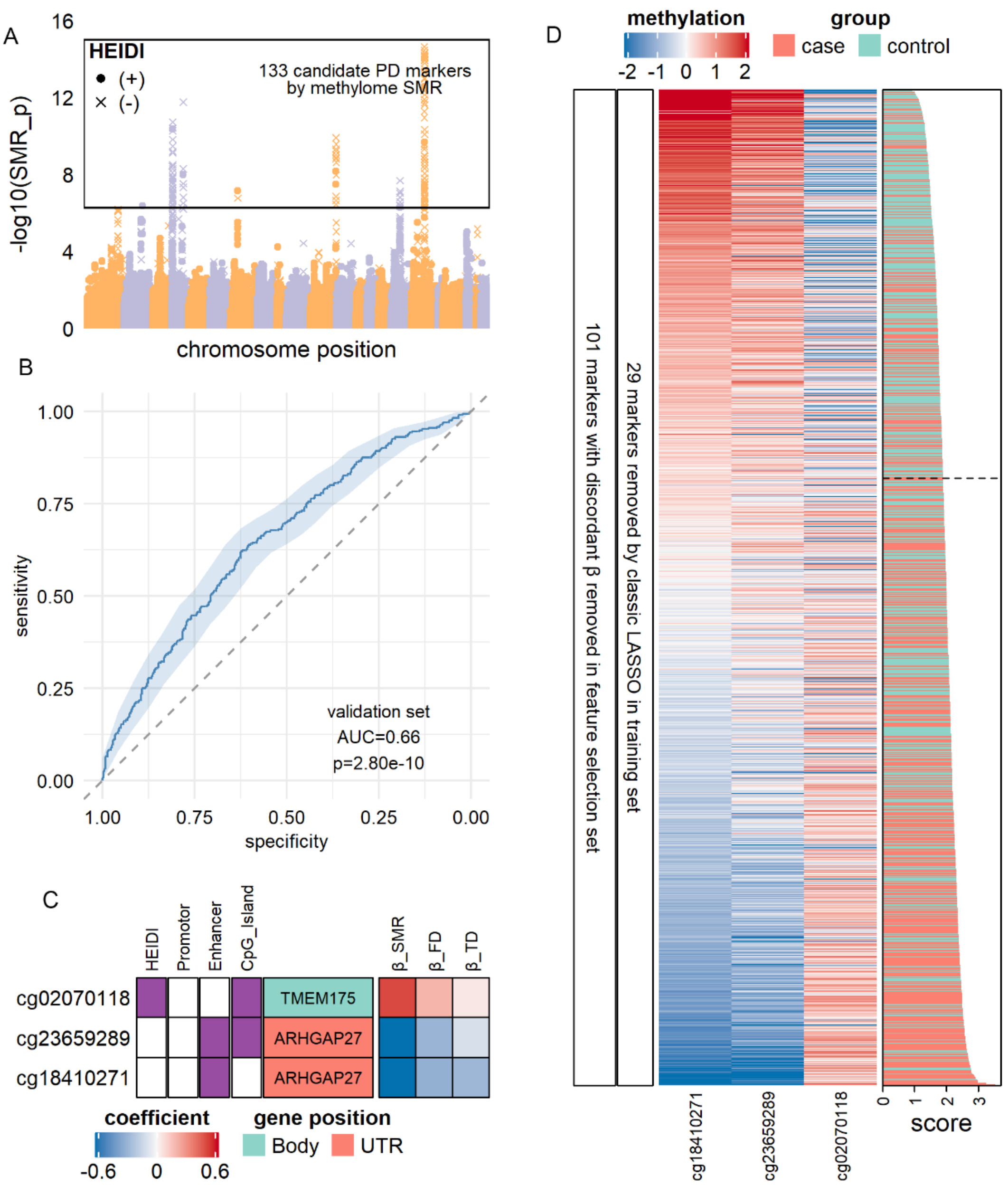

Similar to Figure 3, but for PD.

Supplementary Figure S9 Diagnostic model of BP by RNA markers

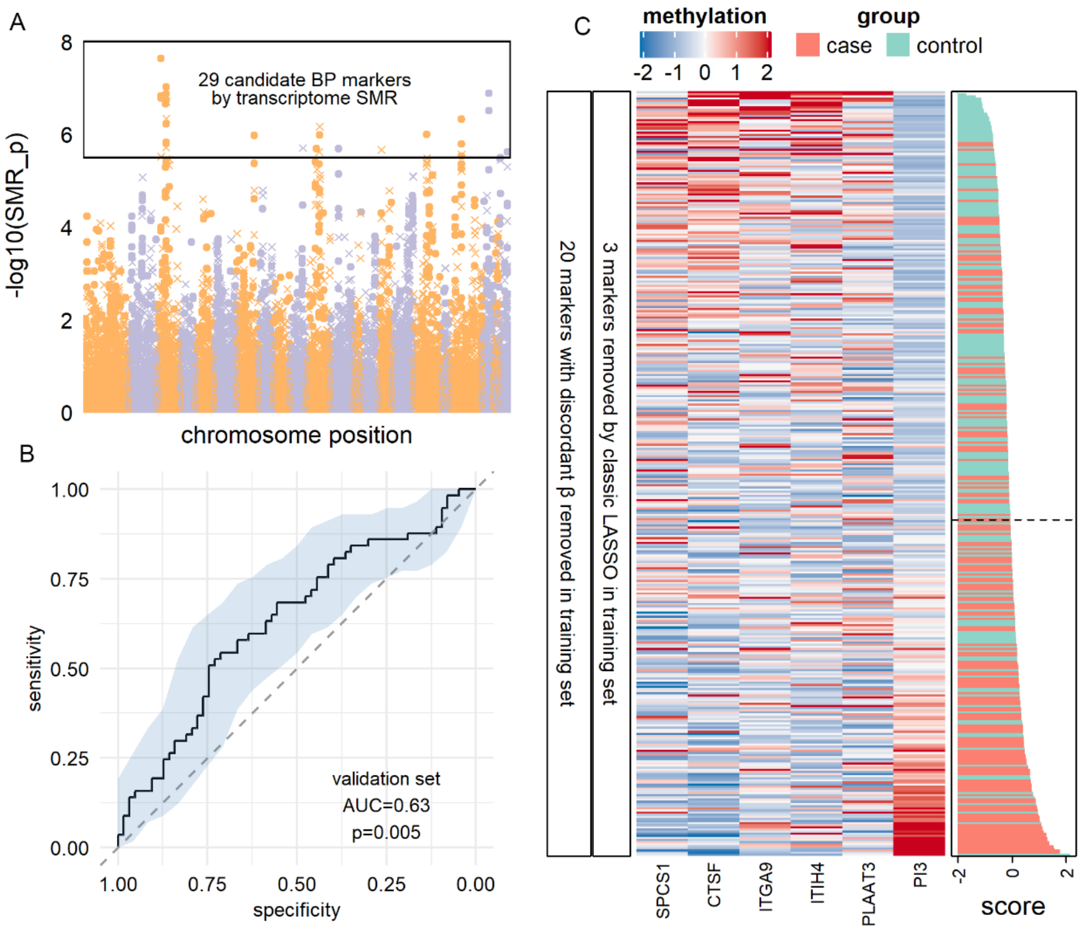

Similar to Figure 4, but for BP RNA markers.

Supplementary Figure S10 Validation of methylation diagnostic markers of AD.

Similar to Figure 3, but for AD.

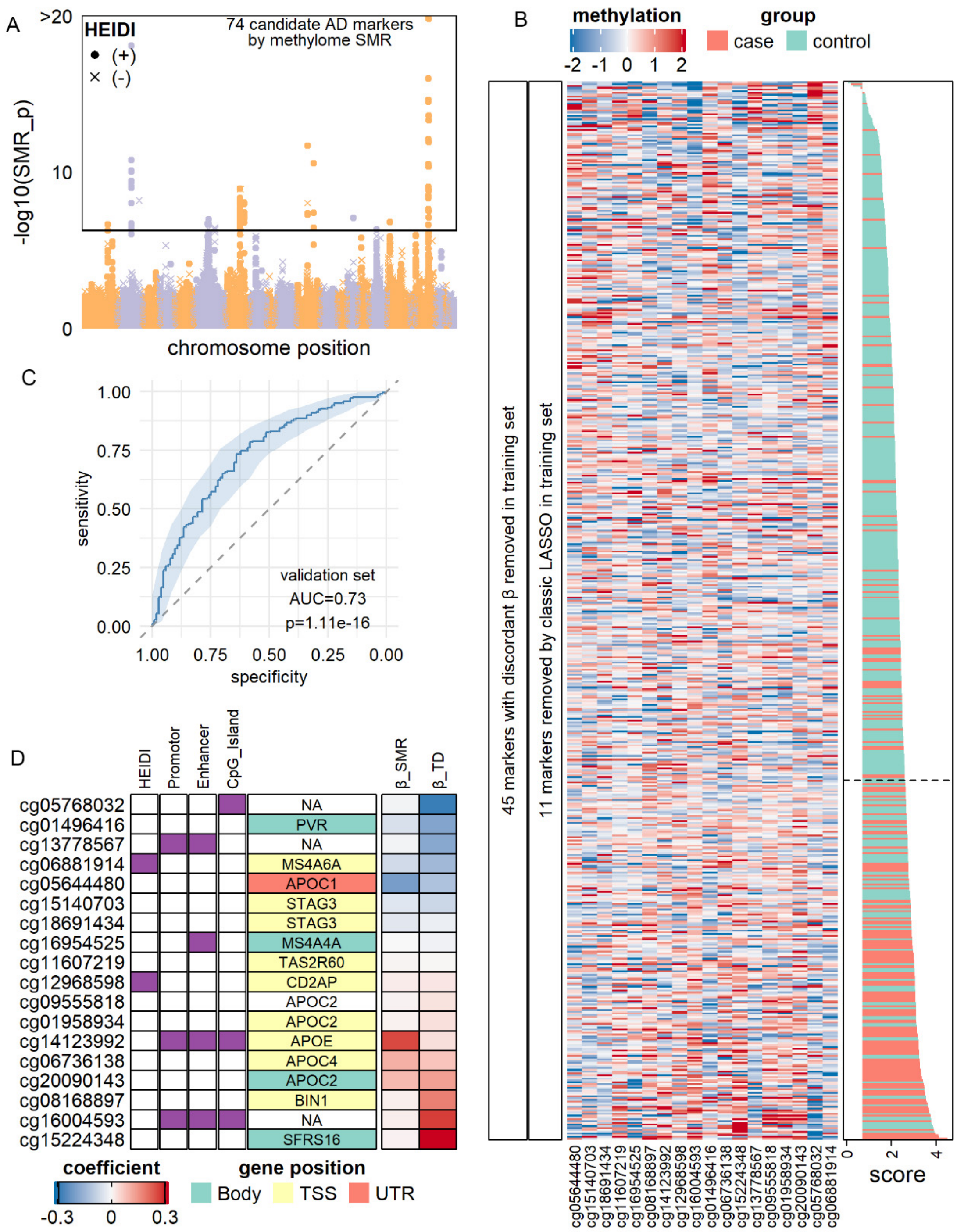

Reference

1. Watanabe K, Stringer S, Frei O, Umićević Mirkov M, de Leeuw C, Polderman TJC, et al. A global overview of pleiotropy and genetic architecture in complex traits. Nat Genet. 2019;51:1339–1348.
2. Hemani G, Zheng J, Elsworth B, Wade KH, Haberland V, Baird D, et al. The MR-base platform supports systematic causal inference across the human phenome. Elife. 2018;7.
3. Leek JT, Johnson WE, Parker HS, Jaffe AE, Storey JD. The sva package for removing batch effects and other unwanted variation in high-throughput experiments. Bioinformatics. 2012;28:882–883.
4. Wu Y, Zeng J, Zhang F, Zhu Z, Qi T, Zheng Z, et al. Integrative analysis of omics summary data reveals putative mechanisms underlying complex traits. Nat Commun. 2018;9:918.
5. Hemani G, Bowden J, Davey Smith G. Evaluating the potential role of pleiotropy in Mendelian randomization studies. Hum Mol Genet. 2018;27:R195–R208.
6. Verbanck M, Chen CY, Neale B, Do R. Detection of widespread horizontal pleiotropy in causal relationships inferred from Mendelian randomization between complex traits and diseases. Nat Genet. 2018;50:693–698.
7. Genovese G, Fromer M, Stahl EA, Ruderfer DM, Chambert K, Landén M, et al. Increased burden of ultra-rare protein-altering variants among 4,877 individuals with schizophrenia. Nat Neurosci. 2016;19:1433–1441.
8. Saunders A, Macosko EZ, Wysoker A, Goldman M, Krienen FM, de Rivera H, et al. Molecular Diversity and Specializations among the Cells of the Adult Mouse Brain. Cell. 2018;174:1015-1030.e16.
9. Skene NG, Grant SGN. Identification of Vulnerable Cell Types in Major Brain Disorders Using Single Cell Transcriptomes and Expression Weighted Cell Type Enrichment. Front Neurosci. 2016;10:16.
10. Yu G, Wang L-G, Han Y, He Q-Y. clusterProfiler: an R Package for Comparing Biological Themes Among Gene Clusters. Omi A J Integr Biol. 2012;16:284–287.
11. Andersson R, Gebhard C, Miguel-Escalada I, Hoof I, Bornholdt J, Boyd M, et al. An atlas of active enhancers across human cell types and tissues. Nature. 2014;507:455–461.
12. ENCODE Project Consortium. An integrated encyclopedia of DNA elements in the human genome. Nature. 2012;489:57–74.
13. Kundaje A, Meuleman W, Ernst J, Bilenky M, Yen A, Heravi-Moussavi A, et al. Integrative analysis of 111 reference human epigenomes. Nature. 2015;518:317–330.
14. Ernst J, Kellis M. ChromHMM: Automating chromatin-state discovery and characterization. Nat Methods. 2012;9:215–216.
15. Nho K, Nudelman K, Allen M, Hodges A, Kim S, Risacher SL, et al. Genome-wide transcriptome analysis identifies novel dysregulated genes implicated in Alzheimer's pathology. Alzheimer's Dement. 2020;16:1213–1223.
16. Krebs CE, Ori APS, Vreeker A, Wu T, Cantor RM, Boks MPM, et al. Whole blood transcriptome analysis in bipolar disorder reveals strong lithium effect. Psychol Med. 2019:1–12.
17. Calligaris R, Banica M, Roncaglia P, Robotti E, Finaurini S, Vlachouli C, et al. Blood transcriptomics of drug-naïve sporadic Parkinson's disease patients. BMC Genomics.

2015;16.
18. Gardiner EJ, Cairns MJ, Liu B, Beveridge NJ, Carr V, Kelly B, et al. Gene expression analysis reveals schizophrenia-associated dysregulation of immune pathways in peripheral blood mononuclear cells. J Psychiatr Res. 2013;47:425–437.
19. Pramparo T, Pierce K, Lombardo M V., Carter Barnes C, Marinero S, Ahrens-Barbeau C, et al. Prediction of Autism by Translation and Immune/Inflammation Coexpressed Genes in Toddlers From Pediatric Community Practices. JAMA Psychiatry. 2015;72:386.
20. Van Eijk KR, De Jong S, Strengman E, Buizer-Voskamp JE, Kahn RS, Boks MP, et al. Identification of schizophrenia-associated loci by combining DNA methylation and gene expression data from whole blood. Eur J Hum Genet. 2015;23:1106–1110.
21. Leday GGR, Vértes PE, Richardson S, Greene JR, Regan T, Khan S, et al. Replicable and Coupled Changes in Innate and Adaptive Immune Gene Expression in Two Case-Control Studies of Blood Microarrays in Major Depressive Disorder. Biol Psychiatry. 2018;83:70–80.
22. Shamir R, Klein C, Amar D, Vollstedt EJ, Bonin M, Usenovic M, et al. Analysis of blood-based gene expression in idiopathic Parkinson disease. Neurology. 2017;89:1676–1683.
23. McCaffrey TA, St. Laurent G, Shtokalo D, Antonets D, Vyatkin Y, Jones D, et al. Biomarker discovery in attention deficit hyperactivity disorder: RNA sequencing of whole blood in discordant twin and case-controlled cohorts. BMC Med Genomics. 2020;13:160.
24. Sood S, Gallagher IJ, Lunnon K, Rullman E, Keohane A, Crossland H, et al. A novel multi-tissue RNA diagnostic of healthy ageing relates to cognitive health status. Genome Biol. 2015;16.
25. Clelland CL, Read LL, Panek LJ, Nadrich RH, Bancroft C, Clelland JD. Utilization of Never-Medicated Bipolar Disorder Patients towards Development and Validation of a Peripheral Biomarker Profile. PLoS One. 2013;8.
26. Gazestani VH, Pramparo T, Nalabolu S, Kellman BP, Murray S, Lopez L, et al. A perturbed gene network containing PI3K–AKT, RAS–ERK and WNT–β-catenin pathways in leukocytes is linked to ASD genetics and symptom severity. Nat Neurosci. 2019;22:1624–1634.
27. Vasanthakumar A, Davis JW, Idler K, Waring JF, Asque E, Riley-Gillis B, et al. Harnessing peripheral DNA methylation differences in the Alzheimer's Disease Neuroimaging Initiative (ADNI) to reveal novel biomarkers of disease. Clin Epigenetics. 2020;12.
28. Osborne L, Clive M, Kimmel M, Gispen F, Guintivano J, Brown T, et al. Replication of epigenetic postpartum depression biomarkers and variation with hormone levels. Neuropsychopharmacology. 2016;41:1648–1658.
29. Sabunciyan S, Maher B, Bahn S, Dickerson F, Yolken RH. Association of DNA methylation with acute mania and inflammatory markers. PLoS One. 2015;10.
30. Vallerga CL, Zhang F, Fowdar J, McRae AF, Qi T, Nabais MF, et al. Analysis of DNA methylation associates the cystine–glutamate antiporter SLC7A11 with risk of Parkinson's disease. Nat Commun. 2020;11.
31. Ratanatharathorn A, Boks MP, Maihofer AX, Aiello AE, Amstadter AB, Ashley-Koch AE, et al. Epigenome-wide association of PTSD from heterogeneous cohorts with a common multi-site analysis pipeline. Am J Med Genet Part B Neuropsychiatr Genet. 2017;174:619–630.

32. Chuang Y-H, Paul KC, Bronstein JM, Bordelon Y, Horvath S, Ritz B. Parkinson's disease is associated with DNA methylation levels in human blood and saliva. Genome Med. 2017;9:76.
33. Lohoff FW, Roy A, Jung J, Longley M, Rosoff DB, Luo A, et al. Epigenome-wide association study and multi-tissue replication of individuals with alcohol use disorder: evidence for abnormal glucocorticoid signaling pathway gene regulation. Mol Psychiatry. 2020:1–14.
34. Booij L, Casey KF, Antunes JM, Szyf M, Joober R, Israël M, et al. DNA methylation in individuals with anorexia nervosa and in matched normal-eater controls: A genome-wide study. Int J Eat Disord. 2015;48:874–882.
35. Hannon E, Dempster E, Viana J, Burrage J, Smith AR, Macdonald R, et al. An integrated genetic-epigenetic analysis of schizophrenia: evidence for co-localization of genetic associations and differential DNA methylation. Genome Biol. 2016;17:176.
